# Synergetic therapy of glioma mediated by a dual delivery system loading α-mangostin and doxorubicin through cell cycle arrest and apoptotic pathways

**DOI:** 10.1038/s41419-020-03133-1

**Published:** 2020-10-28

**Authors:** Wen Nie, Xin Zan, Ting Yu, Mengni Ran, Zehua Hong, Yihong He, Tingting Yang, Yan Ju, Xiang Gao

**Affiliations:** Department of Neurosurgery and Institute of Neurosurgery, State Key Laboratory of Biotherapy and Cancer Center, West China Hospital, West China Medical School, Sichuan University and Collaborative Innovation Center for Biotherapy, Chengdu, 610041 PR China

**Keywords:** Chemotherapy, CNS cancer

## Abstract

Two of the biggest hurdles in the deployment of chemotherapeutics against glioma is a poor drug concentration at the tumor site and serious side effects to normal tissues. Nanocarriers delivering different drugs are considered to be one of the most promising alternatives. In this study, a dual delivery system (methoxy poly(ethylene glycol)-poly(ε-caprolactone) (MPEG-PCL)) loaded with α-mangostin (α-m) and doxorubicin (Dox) was decorated and constructed by self-assembly to determine its ability to treat glioma. Molecular dynamics simulations showed that MPEG-PCL could provide ideal interaction positions for both α-m and Dox, indicating that the two drugs could be loaded into MPEG-PCL. Based on the in vitro results, MPEG-PCL loaded with α-m and Dox (α-m-Dox/M) with a size of 25.68 nm and a potential of −1.51 mV was demonstrated to significantly inhibit the growth and promote apoptosis in Gl261, C6 and U87 cells, and the effects of the combination were better than each compound alone. The mechanisms involved in the suppression of glioma cell growth were blockage of the cell cycle in S phase by inhibition of CDK2/cyclin E1 and promotion of apoptosis through the Bcl-2/Bax pathway. The synergetic effects of α-m-Dox/M effectively inhibited tumor growth and prolonged survival time without toxicity in mouse glioma models by inducing glioma apoptosis, inhibiting glioma proliferation and limiting tumor angiogenesis. In conclusion, a codelivery system was synthesized to deliver α-m and Dox to the glioma, thereby suppressing the development of glioma by the mechanisms of cell cycle arrest and cellular apoptosis, which demonstrated the potential of this system to improve the chemotherapy response of glioma.

## Introduction

Glioma, a neoplasm originating from neuroepithelial cells, accounts for approximately 75% of the malignant primary brain cancers in adults^1,2^. Despite decades of sustained efforts and advances in surgical treatment, radiotherapy and chemotherapy, the current therapies are still limited, leading to a dismal prognosis. After treatment, survival can only be prolonged for several months, and no cure has been achieved. More than half of the patients died within five years after diagnosis and the cancer had a high recurrence rate after treatment^3^. The main obstacles to treatment are the effective dosage of the drugs and the spread of tumors to the surrounding brain tissue, resulting in the formation of distant metastases^4,5^. These clinical results make the study of glioma urgent. In view of the unsatisfactory overall treatment effects, more effective treatment methods are needed to treat malignant glioma.

Doxorubicin (Dox) is an anthracycline drug that has been used as a first-line chemotherapeutic agent for cancer therapy since the 1960s^6^. It has been used to treat soft tissue tumors, lymphoma and solid tumors, including breast cancer, lung cancer and glioma^7–9^. Its mechanism of action is to inhibit DNA synthesis, damage DNA and activate a variety of signaling pathways to promote cellular apoptosis or necrosis^10,11^. However, Dox is also toxic to normal tissues and organs such as the heart and brain^12–14^. In addition, drug exosmosis can also cause side effects, the severity of which depends on the dosage. Due to its toxicity to normal tissues, its clinical applications have been greatly limited. Thus, in recent years, efforts have been made to study Dox delivery systems to deliver drugs and reduce its toxicity and side effects on normal cells^15–17^.

Mangostins are tropical plants, of which xanthones are important secondary metabolites^18^. As an aprenylated xanthone derivative of mangosteen, α-mangostin (α-m) has been shown to possess a variety of pharmacological properties, including anti-inflammatory, neuroprotective and anticancer effects^19–23^. The antineoplastic effects of α-m were first observed against leukemia^24^. Since then, the anticancer effects of α-m have been confirmed in diverse solid tumors, such as colorectal cancer, breast cancer and brain tumors^25–27^. More importantly, studies have shown that α-m can target cancer cells preferentially, which indicates that α-m can effectively avoid the side effects of other chemotherapeutic drugs^28^. In addition, α-m can be combined with chemotherapeutic drugs to treat tumors, thereby enhancing the therapeutic effects and reducing toxicity and side effects^29^. However, α-m is a poorly soluble drug, which limits its applications.

Methoxy poly(ethylene glycol)-poly(ε-caprolactone) (MPEG-PCL), an amphiphilic polymeric carrier, has a core-shell structure in aqueous environments, which is produced by the copolymerization of PCL and PEG^30^. A large number of poorly water-soluble drugs can be encapsulated by MPEG-PCL using a self-assembly method^31^. MPEG-PCL diblock-based microspheres show high drug encapsulation efficiency, a high total drug release rate accompanied by a low initial burst rate and high biocompatibility^32^. Moreover, after systemic administration in vivo, the hydrophilic structure prolongs the circulation of drugs, thereby enhancing their ability to reach the target tissue^33^. Furthermore, due to the low surface charge of the hydrophilic PEG layer, the absorption of plasma proteins decreases, and the absorption of the mononuclear phagocyte system is also reduced^34^. Therefore, the nanoparticles self-assembled by MPEG-PCL have attracted widespread attention for their extensive applications for the delivery of therapeutic drugs.

**Scheme 1:**
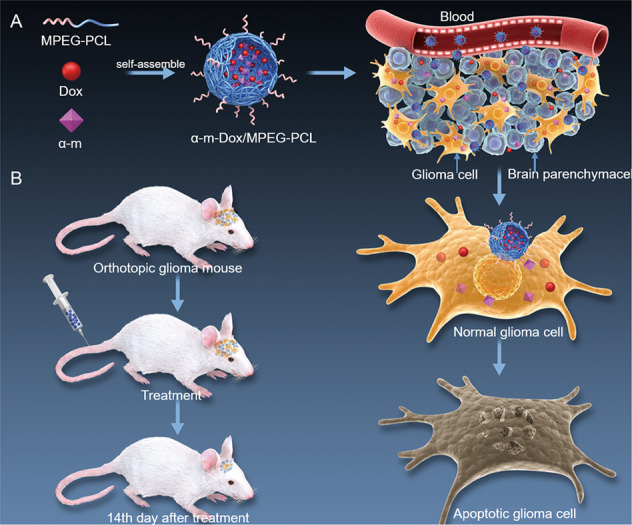
**Schematic representation of the MPEG-PCL carrier for α-m and Dox delivery.** **a** Process of glioma cell apoptosis induced by α-m-Dox/M. **b** An orthotopic implantation glioma model.

Currently, therapies for malignant glioma are limited and accompanied by poor prognosis. In this study, our aim was to use amphiphilic polymeric MPEG-PCL to assemble α-m and Dox for the treatment of glioma and then explore the in vitro and in vivo anticancer effects and mechanism of their synergetic therapy (Scheme 1).

## Materials and methods

### Materials

α-m, Dox, PI, rhodamine 123 (Rh123) and 3-(4,5-dimethylthiazol-2-yl)-2,5-diphenyltetrazolium bromide (MTT) were purchased from Sigma-Aldrich (St. Louis, MO, USA). An Annexin V-FITC/PI Apoptosis Detection Kit and BD Matrigel Basement Membrane Matrix (BD 356234) were purchased from BD Biosciences (Franklin Lake, NJ, USA). The DeadEnd Fluorometric TUNEL System was provided by Promega (Madison, WI, USA). Hematoxylin and eosin (H&E) were purchased from Beyotime (Shanghai, China). Antibodies against CD31 (ab28364) and Ki67 (ab15580) were purchased from Abcam (MA, US). Antibodies used in Western blot were shown in Supplementary materials and methods.

### Cells and animals

The glioma cell lines C6, Gl261 and U87 and human umbilical vein endothelial cells (HUVECs) were purchased from the American Type Culture Collection (ATCC). Primary HUVECs were purchased from ALLCELLS (Shanghai, China). An EGM-2 BulletKit (Lonza, Verviers, Belgium) was used to culture the primary HUVECs. DMEM supplemented with 10% fetal bovine serum (FBS) was used to culture the other cells. All the cells were incubated in a 37 °C incubator with 5% CO_2_. In addition, in vitro data are representative of at least three independent experiments performed in quintuplicate cultures.

BALB/c nude mice and C57BL/6 mice were purchased from Huafukang (Beijing, China). The mice were females aged 6 to 8 weeks that were kept in specific pathogen-free (SPF) conditions. The transgenic zebrafish line was provided by Prof. Shuo Lin (UCLA, Los Angeles, CA) and raised by standard methods as reported previously^35^. The mice used in the animal studies were randomly and blindly divided into five groups. In the subcutaneous glioma models, there were six mice in each group of the Gl261 model (*n* = 6 each group) and seven mice in each group of the C6 model (*n* = 7 each group). In the orthotopic implantation glioma model, there were sixteen mice in each group (*n* = 16 each group). The National Institutes of Health Guide for the Care and Use of Laboratory Animals (NIH Publications No. 8023, revised 1978) was followed for all animal experiments.

### Statistical analysis

Adequate sample size was determined according to previous studies that performed analogous experiments. Variances between groups that were statistically compared were similar. Statistical analysis was performed by GraphPad Prism 7.00. Data analysis was performed by ANOVA and Student’s *t*-test. Kaplan–Meier survival analysis (log-rank test) was used. All data are represented as the mean ± s.e.m. Statistically significant data are marked with *p < 0.05, **p < 0.01, ***p < 0.001.

In addition, the methods of computational simulation, nanocomposite preparation, nanocomposite characterization, in vitro release, MTT assay, flow cytometry, Western blot, scratch assay, transwell assay, tube formation, experimental protocols, optical in vivo imaging, TUNEL assay, histology and drug toxicity assessments are shown in Supplementary materials and methods.

## Results

### Computational molecular dynamics simulation analysis

**Fig. 1:**
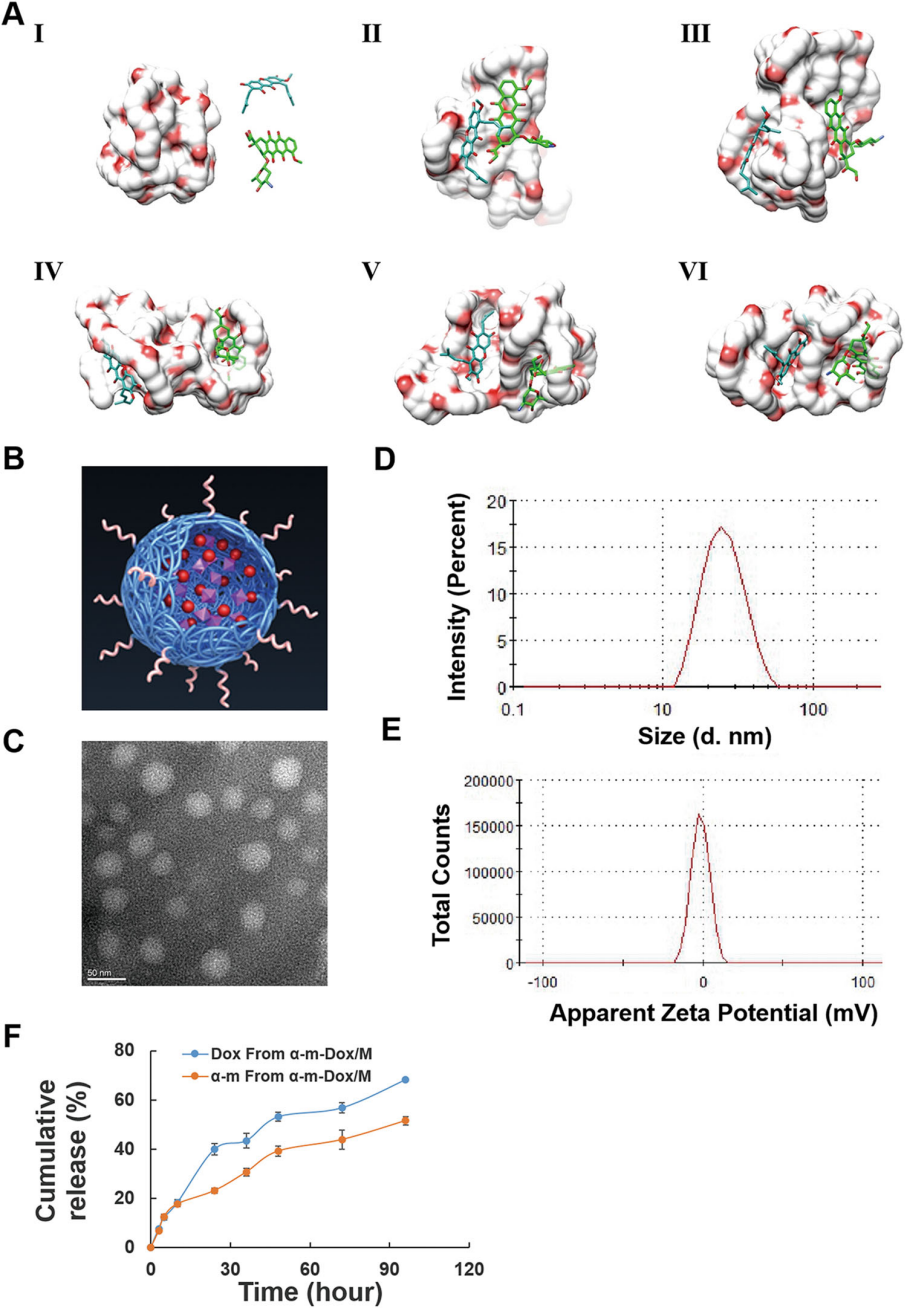
Computational simulation analysis and characterization of nanocomposites. **a** Interaction modes of the MPEG-PCL copolymer, α-m and Dox revealed by Langevin dynamics simulations. The nanocomposite is depicted with a solid surface. (I) The initial conformation of the MPEG-PCL copolymer complexed with α-m and Dox. The left, upper right and lower right represent the MPEG-PCL copolymer, α-m and Dox, respectively. Conformations (II), (III), (IV), (V) and (VI) correspond to snapshots of the nanocomposite collected at 100, 200, 300, 400 and 500 ps, respectively. **b** Structure of the model of α-m-Dox/M. **c** TEM image of α-m-Dox/M. **d** Size distribution spectrum of α-m-Dox/M. **e** Zeta potential of α-m-Dox/M. **f** In vitro release study of α-m-Dox/M.

We used computer simulations to explore the interactions between α-m, Dox and the MPEG-PCL copolymer. As shown in Fig. 1a and Fig. S1, they slowly approached and interacted with each other in an aqueous environment. By changing the location and adjusting the conformation, α-m and Dox found their proper places on MPEG-PCL for combination. Simultaneously, with a dynamic adjustment in the conformation, MPEG-PCL provided suitable binding sites for α-m and Dox. Therefore, a stable system was built through mutual coordination.

### Characterization of the nanocomposites

The structural model of the nanocomposite is displayed in Fig. 1b. The morphology of MPEG-PCL loaded with α-m and Dox (α-m-Dox/M) observed by TEM was a spherical structure with a diameter of approximately 25 nm (Fig. 1c). As shown in Fig. 1d, the nanocomposite had a mean hydrodynamic diameter of 25.68 nm. Moreover, the surface potential of the nanocomposite was negative (−1.51 mV) (Fig. 1e). In addition, the drug loading (DL) and encapsulation efficiency (EE) of the drugs in α-m-Dox/M were 5% and 99.5% for α-m and 5% and 93.5% for Dox, respectively. As shown in Fig. 1f, α-m and Dox were gradually released from α-m-Dox/M, reaching their peak release of 68% and 51% of the total drug over 96 h, respectively.

### Inhibition of cell viability and cell cycle arrest in S phase in vitro

**Fig. 2:**
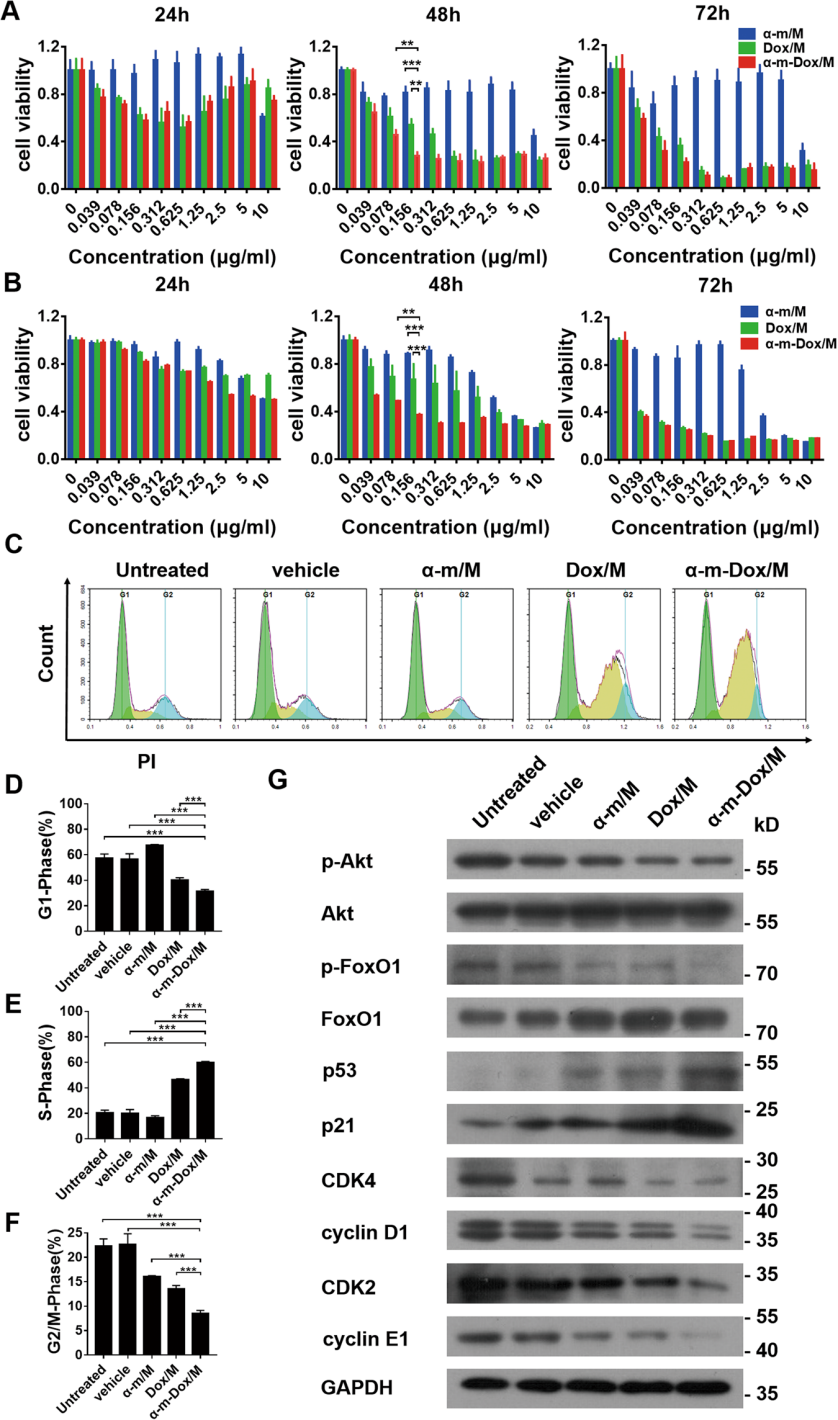
Cell viability study and cell cycle assay. Glioma cells were treated with α-m/M, Dox/M and α-m-Dox/M at different concentrations for 24, 48 and 72 h. The concentrations of the nanocomposites were 0, 0.039, 0.078, 0.156, 0.312, 0.625, 1.25, 2.5, 5 and 10 μg/ml. Then, MTT assays were used to assess cell viability. **a** Gl261 cells. **b** C6 cells. Gl261 cells were incubated with different nanocomposites for 48 h. Then, cells were collected and stained with PI. In addition, cells were collected for Western blot analysis. **c** Cell cycle by flow cytometry. **d** Percent of cells in G1 phase of the cell cycle. **e** Percent of cells in S phase of the cell cycle. **f** Percent of cells in G2/M phase of the cell cycle. **g** Western blot of cell cycle-related proteins. Data are shown as the mean ± s.e.m. and are representative of at least three independent experiments performed in quintuplicate cultures. *p < 0.05, **p < 0.01, ***p < 0.001; ANOVA and Student’s *t*-test.

The MTT assay was utilized to detect the viability of Gl261, C6 and U87 cells after different treatments at different concentrations for 24, 48 and 72 h. The results indicated that a single treatment with Dox/M and combined therapy with α-m-Dox/M inhibited cellular activity in a time-dependent and dose-dependent manner within a certain concentration range (0–0.312 μg/ml). As shown in Fig. 2a, the percent viability of Gl261 cells treated with α-m-Dox/M at a concentration of 0.156 μg/ml for 48 h was approximately 25%, while the viability after single treatment with Dox/M was approximately 55% (α-m-Dox/M vs Dox/M, *p* < 0.001). The combined concentration above was the minimum dose required to achieve the maximum inhibitory effect on cellular activity. Similarly, this result was also found in C6 cells (Fig. 2b). However, with regard to U87 cells, the effect was better with the same concentration after combined treatment for 72 h (Fig. S2). All the results illustrate that therapy with α-m-Dox/M at a low dose (0.156 μg/ml) can inhibit cellular activity more effectively than a single treatment.

To investigate the mechanisms involved in inhibiting the growth of glioma cells, the cell cycle and related proteins were examined. As displayed in Fig. 2c–f, the proportion of cells in S phase after treatment with α-m-Dox/M was 60.27%, while those in the other groups was 20.51% (untreated), 20.04% (vehicle), 16.50% (α-m/M) and 46.35% (Dox/M) (α-m-Dox/M vs Dox/M, *p* < 0.001). Furthermore, compared with single treatment, the expression levels of the cyclin-dependent kinases CDK2, cyclin D1 and cyclin E1 were significantly decreased, while the cyclin-dependent kinase inhibitor (CDKI) p21 and the tumor suppressor p53 were upregulated in the α-m-Dox/M group (Fig. 2g and Fig. S3). Moreover, there was a slight decrease in p-FoxO1/FoxO1 and p-Akt/Akt after treatment with α-m-Dox/M (Fig. S3). Thus, these results suggest that S phase was blocked by α-m-Dox/M through CDKs/cyclins.

### Increased apoptosis in vitro

**Fig. 3:**
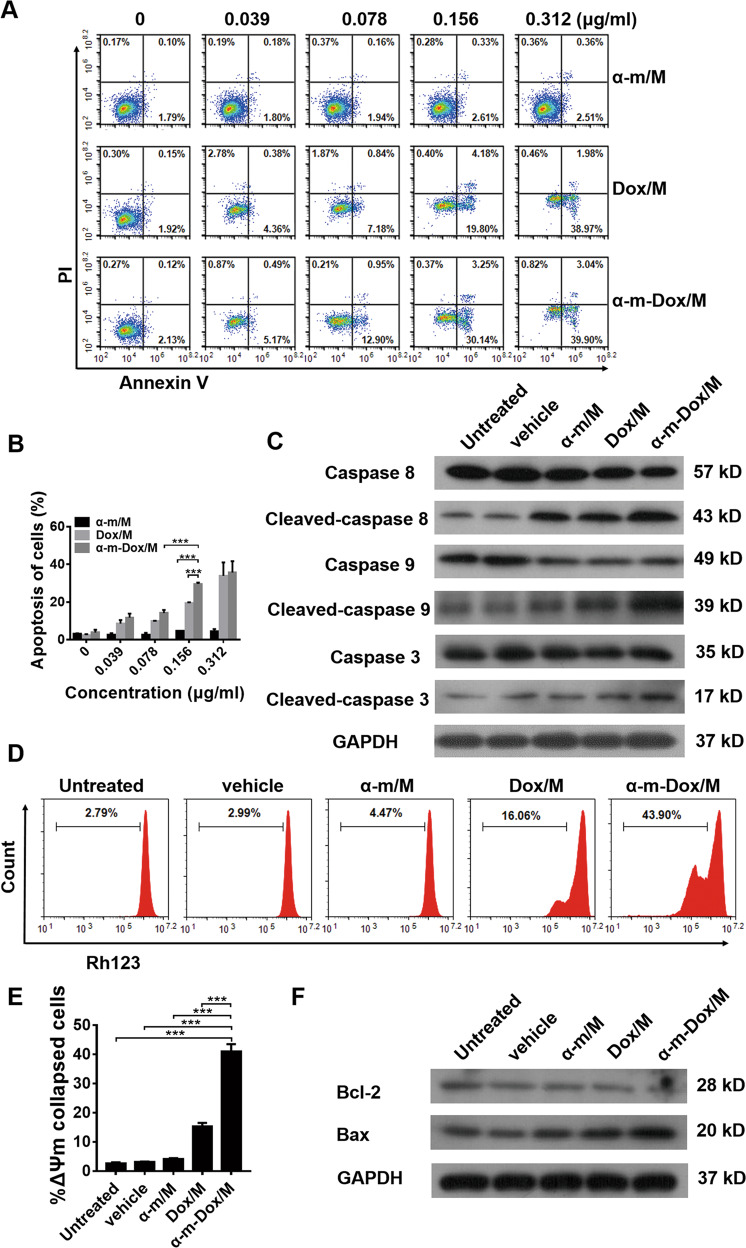
Apoptosis assay. Gl261 cells were incubated with different nanocomposites at different concentrations for 48 h. Cells were collected and stained with Annexin-V and PI. The concentrations of the nanocomposites were 0, 0.039, 0.078, 0.156 and 0.312 μg/ml. (**a, b**) Percent of apoptotic cells. Gl261 cells incubated with different nanocomposites for 48 h were collected for Western blot analysis. **c** Western blot of apoptosis-related proteins. Gl261 cells were incubated with different nanocomposites (0.156 μg/ml) for 48 h. Cells were collected and stained with Rh123. In addition, cells were collected for Western blot analysis. (**d**, **e**) Changes in mitochondrial membrane potential (ΔΨm). **f** Western blot analysis of mitochondrial apoptosis-related proteins. Data are presented as the mean ± s.e.m. and are representative of at least three independent experiments performed in quintuplicate cultures. *p < 0.05, **p < 0.01, ***p < 0.001; ANOVA and Student’s *t*-test.

To further verify the efficacy of the nanocomposites at different concentrations, treated glioma cells were stained with PI and Annexin V-FITC to assess necrosis and apoptosis. As shown in Fig. 3 and Figs. S4, S5, the percent apoptotic cells treated with α-m/M showed only a slight change, which suggests that single treatment with α-m/M has little effect on glioma cells. Dox/M alone and the combined treatment promoted cell apoptosis, and the proportion of cell apoptosis was positively correlated with the dosage of drug. When the concentration was 0.156 μg/ml, 30.14% of the Gl261 cells in the combined treatment group were apoptotic, while that rate was 2.61% in the single α-m/M group and 19.8% in the Dox/M group (*p* < 0.001) (Fig. 3a, b). Analogously, these results were also observed in C6 (Fig. S4) and U87 cells (Fig. S5). Moreover, the apoptosis-related proteins caspase 8, caspase 9 and caspase 3 were more highly activated in cells treated with α-m-Dox/M than in cells treated with a single treatment (Fig. 3c and Fig. S3). These results indicate that α-m-Dox/M promotes apoptosis of glioma cells.

Changes in the mitochondrial membrane potential (ΔΨm) were considered relevant to the intrinsic apoptotic pathway. In Fig. 3d, e, the percentage of ΔΨm collapsed cells treated with α-m-Dox/M was 43.9%, while those in other groups was 2.79% (untreated), 2.99% (vehicle), 4.47% (α-m/M) and 16.06% (Dox/M) (α-m-Dox/M vs Dox/M, *p* < 0.001). In addition, we observed significant downregulation of the antiapoptotic protein Bcl-2 and upregulation of the proapoptotic protein Bax in glioma cells treated with α-m-Dox/M (Fig. 3f and Fig. S3). These results demonstrate that the intrinsic apoptotic pathway is involved in the antitumor effects of α-m-Dox/M.

### Inhibition of migration and tube formation in vitro

In order to reveal the effects of α-m-Dox/M on HUVEC migration capacity in vitro, scratch and transwell assays were performed. As shown in Fig. S6a, there was a significant inhibitory effect from α-m-Dox/M on the HUVEC migration rate after 48 h compared to the other treatments (α-m-Dox/M vs Dox/M, *p* < 0.001). In addition, the results of the transwell assay were consistent with those of the scratch assay (Fig. S6b). On the other hand, tube formation of the primary HUVECs was significantly suppressed after treatment with α-m-Dox/M (Fig. S6c). Thus, the data showed that α-m-Dox/M could blunt the migration rate and tube formation in HUVECs.

### Antitumor properties in a transgenic zebrafish tumor model

To directly and quickly observe the therapeutic effects of the drugs in vivo, a FLK-1 promoter EGFP transgenic (Tg(FLK-1:EGFP)) zebrafish line was used for the antitumor evaluation. Endothelial cells from Tg(FLK-1:EGFP) zebrafish showed green fluorescence. The tumors in zebrafish showed red fluorescence. As shown in Fig. S7, the tumor volumes in the combined treatment group were significantly smaller than those in the single treatment groups. Therefore, we conclude that treatment with α-m-Dox/M inhibits tumor growth in a zebrafish model more effectively than single treatment.

### Antitumor properties in subcutaneous glioma models

**Fig. 4:**
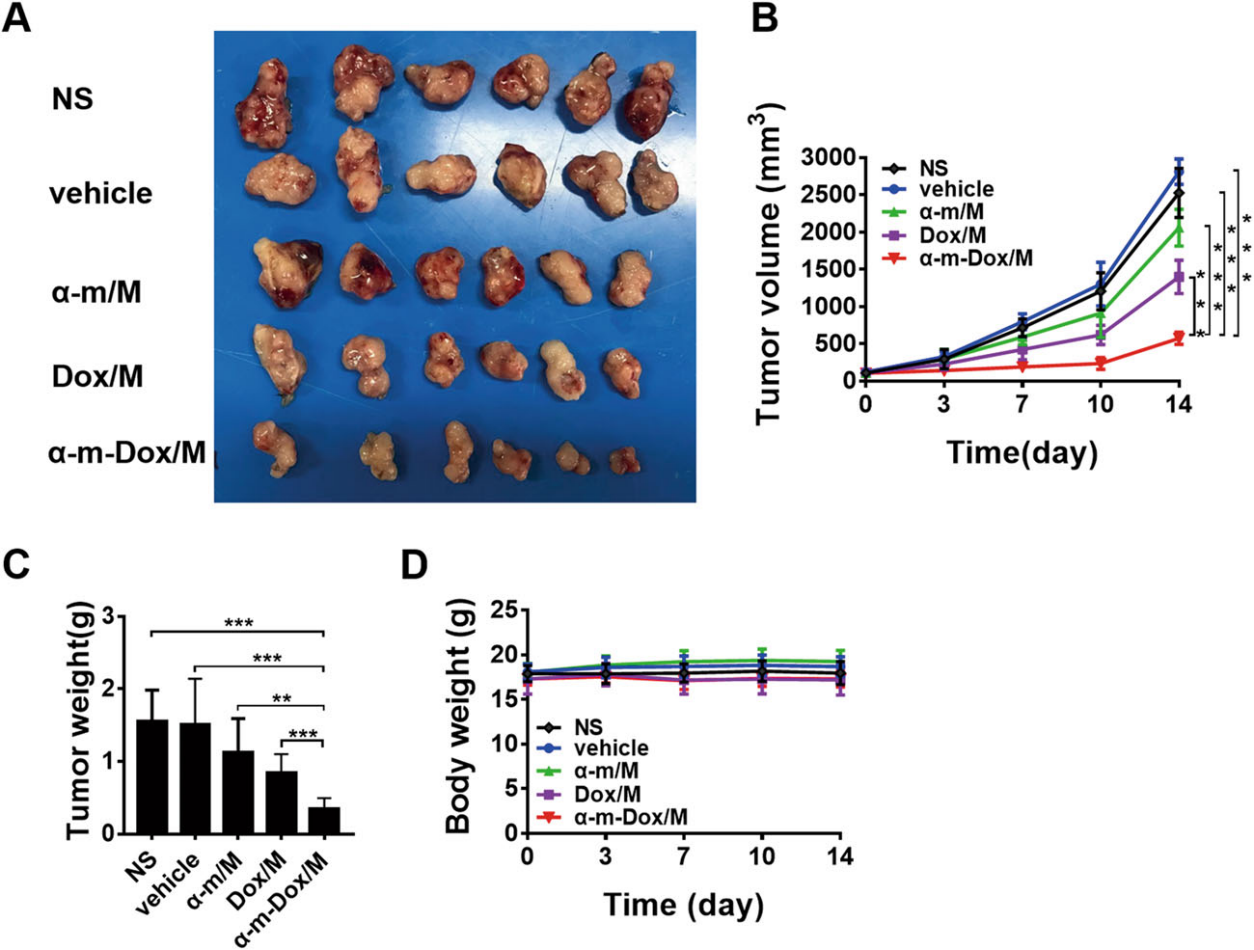
Antitumor effects in the subcutaneous Gl261 glioma model. C57BL/6 mice were subcutaneously inoculated with 1×10^6^ Gl261 cells and treated with NS, vehicle, Dox/M, α-m/M or α-m-Dox/M once every three days. **a** Images of the tumors from different groups. **b** Tumor growth curves. **c** Tumor weights. **d** Body weights. Data are representative of at least three independent experiments. *n* = 6; *p < 0.05, **p < 0.01, ***p < 0.001; ANOVA and Student’s *t*-test.

**Fig. 5:**
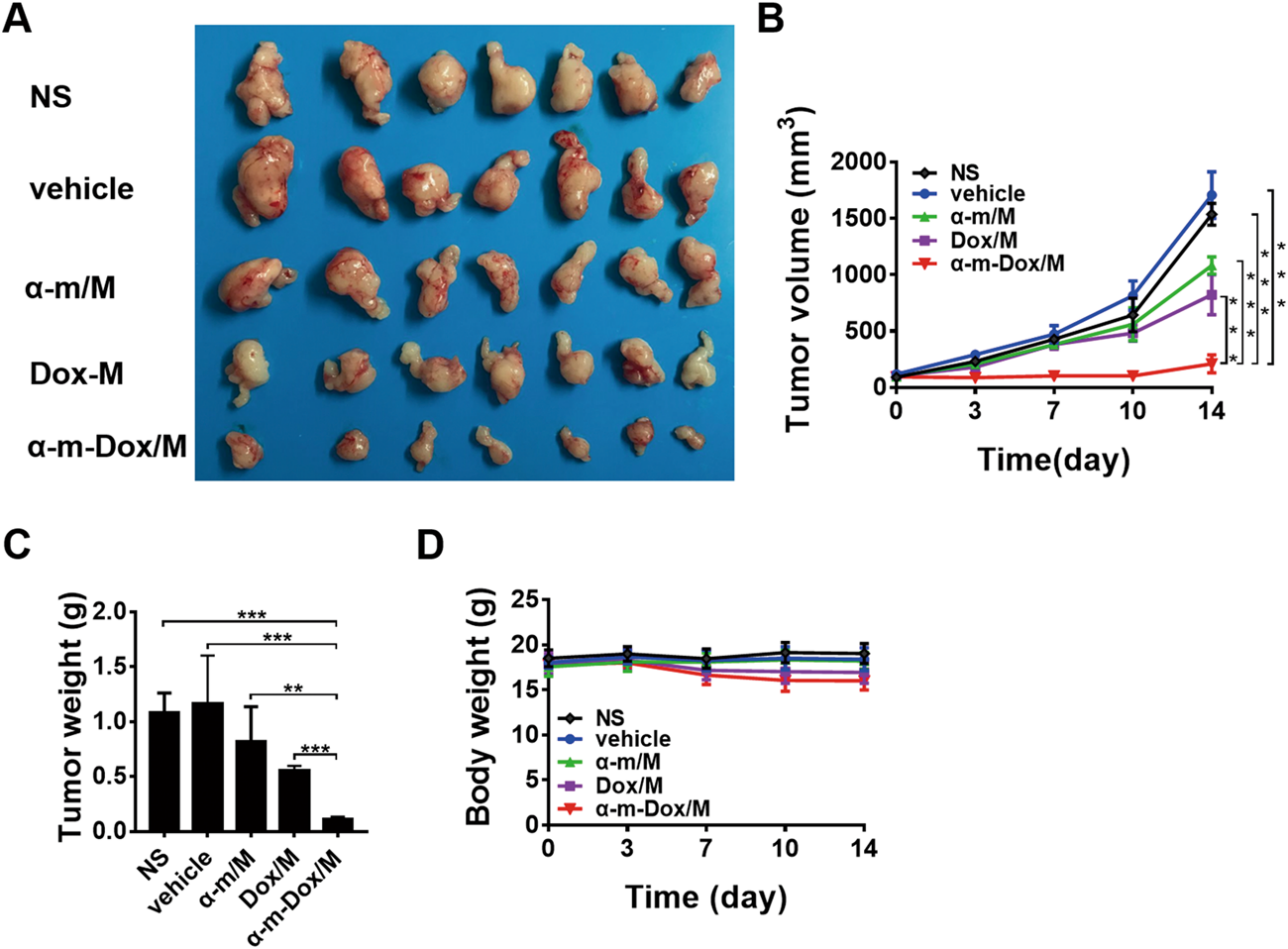
Antitumor effects in the subcutaneous C6 glioma model. Nude BALB/c mice were subcutaneously inoculated with 1×10^7^ C6 cells and treated with NS, vehicle, Dox/M, α-m/M or α-m-Dox/M once every three days. **a** Images of the tumors from different groups. **b** Tumor growth curves. **c** Tumor weights. **d** Body weights. Data are representative of at least three independent experiments. *n* = 7; *p < 0.05, **p < 0.01, ***p < 0.001; ANOVA and Student’s *t*-test.

In vivo, the antitumor activity of the nanocomposites was investigated in Gl261 and C6 subcutaneous models. In the Gl261 model, treatment with α-m-Dox/M performed the best in suppressing tumor growth among all groups (Fig. 4). On the 14th day after treatment, the average tumor volume in the combined group was 500 cm^3^ (Fig. 4a, b), and the average weight of the tumors was 0.7 g (Fig. 4c), which was the smallest and lightest of all five groups (*p* < 0.001). Similar results were observed from the C6 model (Fig. 5). In addition, there was no obvious decrease in body weight in Fig. 4d and Fig. 5d (*p* > 0.05). Thus, we concluded that treatment with α-m-Dox/M contributes more to the inhibition of tumor growth compared with a single treatment.

### Antitumor and prolonged survival properties in an orthotopic implantation glioma model

**Fig. 6:**
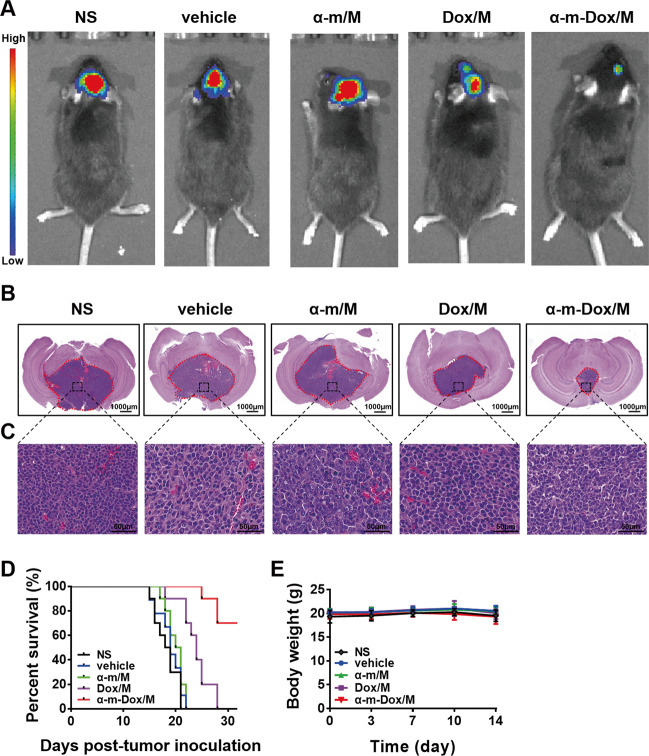
Antitumor effects in an orthotopic implantation glioma model. C57BL/6 mice were inoculated with 1 × 10^3^ Gl261 cells by intracranial implantation and treated with NS, vehicle, Dox/M, α-m/M or α-m-Dox/M intravenously once every three days. **a** Optical in vivo imaging (*n* = 6). **b** H&E staining of the brain. **c** Enlargement of the corresponding position of the tumor. **d** Survival curves of the different groups (*n* = 10). **e** Body weights. Data are representative of at least three independent experiments. *p < 0.05, **p < 0.01, ***p < 0.001; ANOVA, Student’s *t*-test and Kaplan–Meier survival analysis (log-rank test).

As shown in Fig. 6a, b, the combined therapy of α-m-Dox/M prevented tumor progression more efficiently than single treatment since the fluorescence intensity from the tumors in the combination group was the weakest among all groups and the H&E staining of brain tissues displayed the smallest tumor volume. The images in Fig. 6c are enlargements of the tumor sites. The percent of Ki67-positive tumor cells was less than 10% in the α-m-Dox/M group and over 80% in the other groups (*p* < 0.001) (Fig. S8). In addition, by the 30th day after inoculation, all mice treated with a single treatment died, but the survival rate of the mice in the α-m-Dox/M group was 70% (*p* < 0.01) (Fig. 6d). There was no difference in body weight among the groups (*p* > 0.05) (Fig. 6e). These results indicate that treatment with α-m-Dox/M effectively inhibits the growth of glioma in vivo and prolongs the lifetime of the mice.

### Inhibition of cell proliferation and angiogenesis and promotion of cell apoptosis in vivo

**Fig. 7:**
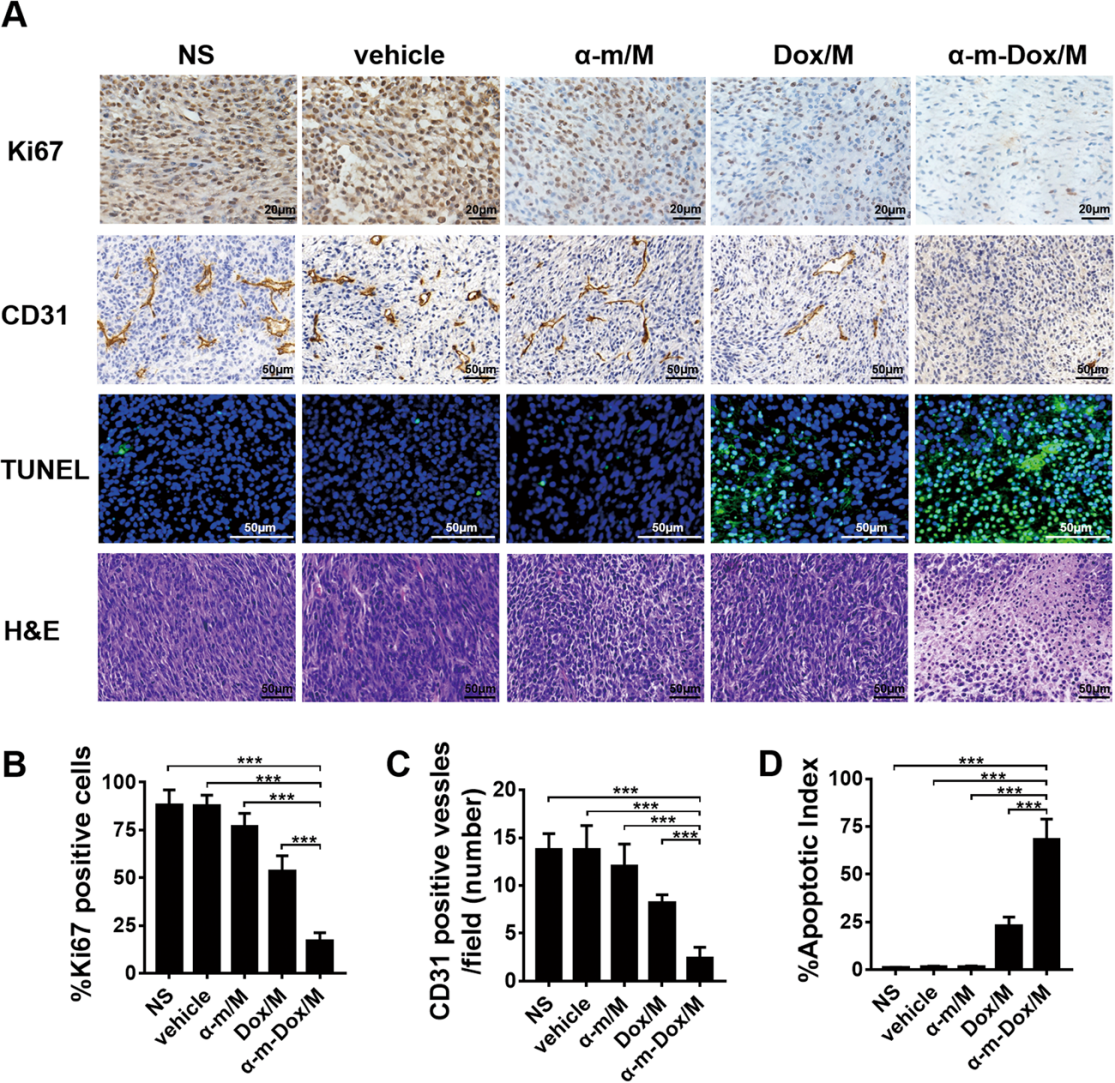
Proliferation, angiogenesis and apoptosis assays in vivo. **a** Ki67 staining was performed in tumor tissue sections of different groups to visualize tumor proliferation. CD31 staining was applied to analyze tumor angiogenesis. TUNEL staining was conducted to study tumor apoptosis. H&E staining was used to observe cell morphology. **b** The average percentage of Ki67-positive cells. **c** The average percentage of CD31-positive vessels. **d** The average percentage of TUNEL-positive cells. The tumor specimens were from the C6 subcutaneous model. Data are representative of at least three independent experiments. *n* = 5; *p < 0.05, **p < 0.01, ***p < 0.001; ANOVA and Student’s *t*-test.

The proliferation of tumor tissues was evaluated by immunohistochemical staining with the Ki-67 antibody, and representative images are displayed in Fig. 7a, b. The average percentage of Ki-67-positive cells in the α-m-Dox/M group (16.8%) was lower than that in the other groups (more than 50%). Angiogenesis was detected by immunohistochemical staining with CD31. As shown in Fig. 7a, c, the number of vessels in the α-m-Dox/M group was the lowest (*p* < 0.001). TUNEL apoptotic staining was used to assess apoptosis in vivo. As shown in Fig. 7a, d, the percentage of the apoptosis index in the α-m-Dox/M group was much higher than that in the other groups (*p* < 0.001). The tumor specimens in Fig. 7 were from the C6 subcutaneous model. Therefore, it was concluded that α-m-Dox/M therapy can effectively inhibit cell proliferation and angiogenesis and promote apoptosis in vivo.

### Safety assessment

All vital organs presented normal histomorphologies (Fig. S9). As shown in Fig. S10 and Fig. S11, the serological biochemistry parameter analysis and complete blood count (CBC) were in the normal range. These results suggest that neither the drugs nor the MPEG-PCL materials are toxic in vivo.

## Discussion

Glioma is a common malignant primary brain tumor with poor prognosis, including death and a high recurrence rate despite decades of sustained efforts and advancements in therapies^3^. Recently, malignant glioma has faced therapeutic barriers, including traditional chemotherapy limitations due to permeability, selectivity and side effects^36^. In this study, MPEG-PCL nanoparticles were synthesized and used as a drug-releasable carrier to deliver both α-m and Dox to treat glioma.

Traditional chemotherapeutic drugs have been demonstrated to have nonspecific cytotoxicity, which causes damage to normal cells^37^. Therefore, various experiments have been constantly performed to develop and improve effective drug delivery systems to minimize drug toxicity and pursue the effects of drugs directly on the tumor as much as possible. In this study, MPEG-PCL was applied to encapsulate and deliver different drugs, which reduced the dosage of each drug to limit the side effects and achieved a better synergistic treatment effect. The constructed nanocomposite has good water solubility and is small in size, which facilitates its passive diffusion through tumor interendothelial junctions. Due to its low surface potential, the nanocomposite is stable in the blood circulation, degradation is slow and the drugs can be continuously released for effective drug accumulation in the tumor. At the same time, a negative charge on the surface of the nanocomposite can reduce its own toxicity. Thus, this delivery system is a perfect carrier. While providing a therapy for cancer, Dox is toxic to the major organs, especially its fatal cardiotoxicity, which limits its dosage and leads to unsatisfactory monotherapy. Similarly, α-m has been reported to promote microbial dysbiosis at high doses, leading to serious inflammation, ulceration and epithelial cell proliferation in the colon^38^. Hence, this delivery system is capable of effectively encapsulating α-m and Dox, overcoming the water-insoluble characteristics of the two drugs, achieving sustained drug release and avoiding the side effects of high-dose single-agent treatment, making it one of the best choices for treating glioma.

In this study, combined therapy with α-m and Dox via the MPEG-PCL delivery system was shown to inhibit the growth of glioma cells in vitro by blocking the cell cycle and promoting apoptosis. First, misregulation of the cell cycle contributes greatly to the abnormal proliferation of cancer cells. Our study suggested that the blockage of the cell cycle in S phase induced by α-m-Dox/M contributed to the suppression of glioma cell proliferative activity by upregulating the expression of p21 and p53 and downregulating the expression of CDK2, cyclin E1, cyclin D1, p-FoxO1/FoxO1 and p-Akt/Akt. Akt has been reported to modulate cell proliferation and the cell cycle by directly targeting p21 and indirectly regulating cyclins and the tumor suppressor p53 through FoxOs^39–41^. FoxO1 is inactivated by Akt during the cell survival regulation and plays a significant role in G1/S arrest by upregulating CDKIs and consequently attenuating CDKs^40,42^. In addition, p53 mainly regulates p21 to maintain homeostasis between cell survival and death, whereas frequent inactivation of p53 in various cancer cells results in the infinite proliferation of cells^43,44^. Eventually, p21 greatly contributes to adjusting the S phase of the cell cycle by suppressing the kinase activity of CDK/cyclin complexes^45^. Therefore, the synergistic treatment of α-m-Dox/M could cause glioma cell cycle arrest in the S phase via CDKs/cyclins, which was demonstrated in our study. When cellular cyclin is arrested at G1/S phase, the apoptosis pathway is triggered. In this study, the mitochondria-dependent apoptosis pathway was involved. Our results showed that synergistic treatment induced drastic changes in the ΔΨm. Furthermore, significant downregulation of Bcl-2 and upregulation of Bax were observed in glioma cells treated with α-m-Dox/M, which may be mediated by the p53 pathway because of the elevated level of the p53 protein^46^. Bcl-2 is an antiapoptotic protein, whose properties are opposed by Bax, and the balance between these proteins is significant for cell apoptosis or survival^10,47^. Theoretically, an altered ratio of Bcl-2/Bax causes downstream activation of caspase cascades (such as caspase 8), ultimately leading to the activation of cleaved caspase 3^48–50^, which was observed in glioma cells after treatment with α-m-Dox/M. It is evident that treatment with α-m-Dox/M inhibits tumor progression through S phase cell cycle arrest and promoting apoptosis via the Bcl-2/Bax pathway.

In vivo, α-m-Dox/M effectively interfered with glioma progression and prolonged the mouse survival time. As reported, α-m and Dox have been shown to inhibit or retard the growth of a series of tumor cells^7,20^, which is consistent with our experimental results, but our results indicate that the combination therapy has a better antitumor effect. Furthermore, it is worth noting that mangostins preferentially target tumor cells rather than normal cells, suggesting that they may avoid the side effects of traditional chemotherapy drugs^29,51^. α-m can act synergistically with traditional chemotherapy drugs, enhancing their efficacy while reducing the side effects on normal tissues. It has been reported that lower concentrations of α-m work synergistically with 5-FU to inhibit the growth of colon cancer, thereby reducing the dosage of 5-FU needed to decrease its systemic side effects^52^. Additionally, α-m prevented normal renal epithelial cells from undergoing apoptosis induced by cisplatin by attenuating oxidative stress and inflammatory cytokines^51^. Importantly, α-m protected against Dox-induced neurotoxicity by regulating the expression of apoptosis-related proteins and ameliorating oxidative damage^53^. On the other hand, Dox has also been reported to act synergistically with other drugs to cure cancers, enhancing their efficacy and reducing their side effects^54^. It has been reported that combined therapy with Dox and curcumin effectively cures head and neck cancer and glioma with fewer side effects^55,56^. Above all, polytherapy with two drugs can lower the dosage and achieve better efficacy, thereby reducing the side effects caused by large doses of monotherapy on normal tissues, which are consistent with our experimental results. In addition, the antitumor mechanism in vivo was that α-m-Dox/M suppressed angiogenesis, induced apoptosis and inhibited the proliferation of tumor cells. Angiogenesis is necessary for tumor proliferation and migration. Once the blood vessels are blocked, the tumor will become hypoxic and oligotrophic, which will cause the proliferation and migration of tumor cells to decrease, leading to apoptosis^57^. Therefore, effective inhibition of angiogenesis may provide a crucial strategy for halting the process of carcinogenesis. Therefore, the synergistic therapy of α-m-Dox/M can efficiently inhibit glioma growth and prolong mouse survival time with a lower dosage and reduced toxicity by limiting angiogenesis, promoting apoptosis and inhibiting proliferation.

In conclusion, α-m-Dox/M was synthesized by self-assembly for the delivery of α-m and Dox to tumor cells, thereby inhibiting the growth of glioma cells by blocking cells in S phase via CDKs/cyclins and promoting cell apoptosis via the Bcl-2/Bax pathway in vitro, suppressing glioma development and prolonging mouse survival time with minimal toxicity by reducing angiogenesis, and promoting apoptosis and inhibiting proliferation of tumor cells in vivo. The combined antitumor effects of α-m-Dox/M and its mechanisms were confirmed in gliomas. Therefore, α-m-Dox/M may be a perfect candidate for the clinical treatment of gliomas.

## Supplementary information

Supplementary materials and methods

Supplementary figure legends

Supplementary figure 1

Supplementary figure 2

Supplementary figure 3

Supplementary figure 4

Supplementary figure 5

Supplementary figure 6

Supplementary figure 7

Supplementary figure 8

Supplementary figure 9

Supplementary figure 10

Supplementary figure 11
