# Supplementary material for: Synergetic therapy of glioma mediated by a dual delivery system loading α-mangostin and doxorubicin through cell cycle arrest and apoptotic pathways
Source: Cell Death Dis. 2020 Oct 28;11(10):928. doi: 10.1038/s41419-020-03133-1 (PMC7595144; doi:10.1038/s41419-020-03133-1)
Supplement: Supplementary file 1 — Supplementary materials and methods [file 41419_2020_3133_MOESM1_ESM.doc]

**Supplementary materials and methods**

**Materials**

Antibodies against Akt (#4691), phospho-Akt (#4060) (p-Akt), Bcl-2 (#3498), Bax (#2772), caspase 3 (#9662), cleaved caspase 3 (#9661), caspase 8 (#4790), cleaved caspase 8 (#9429), caspase 9 (#9508), cleaved caspase 9 (#9509), CDK2 (#2546), CDK4 (#12790), cyclin D1 (#2978), cyclin E1 (#4129), FoxO1 (#2880), phospho-FoxO1 (#9461) (p-FoxO1), p21 (#64016), p53 (#2527), and GAPDH (#97166) were purchased from Cell Signaling Technology (Boston, MA, USA).

**Computational simulation**

Molecules of α-m, Dox and copolymer MPEG-PCL were constructed and further optimized as reported. The interactions among α-m, Dox and MPEG-PCL, under aqueous circumstances, were explored using molecular dynamics simulations. In HyperChem workspace (HyperChem Professional 8.0, Hypercube, Inc., Gainesville, FL, USA), α-m and Dox were randomly docked to the copolymer MPEG-PCL, by which initial structure of nanocomposite was built. Then, Langevin dynamics simulations were performed in order to investigate the interactions among α-m, Dox and copolymer MPEG-PCL. During the simulation, various parameters were set as reported.

**Preparation of nanocomposite**

MPEG (2000)-PCL (2000) diblock copolymer with a molecular weight of 4000 was synthesized by ring opening of ε-caprolactone, which was initiated by MPEG. Briefly, MPEG and ε-CL were introduced into a dry glass ampoule under a nitrogen atmosphere. Sn(Oct)_2_ was then added into the reaction vessel under mild agitation, and the reaction system was kept at 130℃ for 6 h. The purified MPEG-PCL copolymer was kept in a desiccator before further use.

MPEG-PCL, MPEG-PCL loading single α-m (α-m/M), MPEG-PCL loading single Dox (Dox/M), MPEG-PCL loading both α-m and Dox (α-m-Dox/M) were prepared by a self-assembly method. Briefly, 5 mg of α-m and 90 mg of MPEG-PCL diblock copolymer were dissolved in 2 ml of acetone solution, followed by evaporation under reduced pressure in a rotary evaporator at 55°C. Water was then added to the above polymer and drug mixture, allowing self-assembly of α-m and MPEG-PCL, creating core-shell-structured α-m/M micelles with core-encapsulated α-m. α-m-Dox/M micelles were then prepared using a pH-induced self-assembly method. Briefly, 1.4 ml of α-m/M micelles prepared in the previous step was placed into a tube, and 0.2 ml of phosphate-buffered solution (10×, pH 7.4) was added to this solution under stirring. Next, 0.4 ml of Dox aqueous solution (5 mg/ml) was added to the above solution. Because Dox has low solubility in phosphate-buffered solution at pH 7.4, it was able to self-assemble into the hydrophobic core of the MPEG-PCL micelle. Twenty minutes later, α-m-Dox/M micelles were obtained. The α-m/M, Dox/M, and empty MPEG-PCL micelle formulations were prepared in the same way as the α-m-Dox/M micelles, but without α-m or Dox in the mixtures.

A high performance liquid chromatography (HPLC) method was performed to calculate encapsulation efficiency (EE) and drug loading (DL) of α-m/M, Dox/M and α-m-Dox/M. DL and EE of α-m-Dox/M were determined by HPLC method. Briefly, 10 mg lyophilized α-m-Dox/M micelles were dissolved in 0.1 ml of methanol. The solvent delivery system was equipped with a plus auto-sampler and a column heater. Detection was conducted on a Waters 2966 detector. Chromatographic separations were performed on a reversed-phase C 18 column (4.6×150 nm, 5 μm, Sunfre Analysis column), and the column temperature was kept at 28 °C. Methanol-water (70/30, v/v) was used at a ﬂow rate of 1 ml/min as eluent. The EE and DL of each micelle were calculated as follows:

$\text{DL}=\frac{\text{Drug}}{\text{Polymer+Drug}}\times100\%$ (1)

$\text{EE}=\frac{\text{Experimental drug loading}}{\text{Theoretical drug loading}}\times100\%$ (2)

**Characterization of the nanocomposite**

The morphologies of nanocomposite were observed under transmission electron microscopy (TEM, Hitachi H600, Japan). Six visual fields per sample were randomly selected for detection. The average size and zeta potential of the nanocomposite were measured by a Zetasizer NanoZS (Malvern Instruments, Ltd., Worcestershire, UK).

**In vitro release**

To determine the release kinetics of α-m and Dox from α-m-Dox/M nanocomposites, 0.5 ml of α-m-Dox/M nanocomposites was placed in a dialysis bag (molecular weight cutoff, 3.5 kDa) and then incubated in 30 ml of phosphate buffer solution (PBS, pH 7.4) containing Tween-80 (0.5% w/w) at 37 ℃ with gentle shaking. At pre-determined time points, the incubation medium was replaced with fresh one. The amount of released α-m and Dox in the incubation medium was quantified by determining the absorbance at 230 nm using HPLC.

**MTT assay**

MTT assay was used to detect cell viability. Cells were seeded into 96-well plates and incubated overnight. 6000 Gl261 cells (8000 C6 cells and 8000 U87 cells) were seeded into each well of 96-well plate overnight. Then, the cells were treated with different drugs for 24 h. 4000 Gl261 cells (6000 C6 cells and 6000 U87 cells) were seeded for treatment of 48 h. 2000 Gl261 cells (4000 C6 cells and 4000 U87 cells) were seeded for treatment of 72 h. Cells were treated with different drugs including α-m/M, Dox/M and α-m-Dox/M. The concentrations of α-m-Dox/M were as follows: 0, 0.039, 0.078, 0.156, 0.312, 0.625, 1.25, 2.5, 5, 10 μg/ml, as the same with concentrations of α-m/M and Dox/M. After 24 h ,48 h and 72 h of treatment, MTT solution (0.2 mg/ml) was added and co-incubated with cells for 2 h. Then, assess the ability of cells to metabolize MTT by spectrophotometry.

**Flow cytometry**

Annexin V-FITC/PI staining was used to detect cell necrosis and apoptosis by flow cytometry. Glioma cells were cultured overnight in 6-well plates, after which cells were co-incubated with MPEG-PCL loading single α-m (α-m/M), MPEG-PCL loading single Dox (Dox/M) and MPEG-PCL loading both α-m and Dox (α-m-Dox/M). After 48 h, we collected cells and performed Annexin V-FITC/PI assay according to the manufacturer’s instruction.

Mitochondrial membrane potential was detected by Rhodamine 123 (Rh123) staining. Cells were cultured overnight in 12-well plates and then co-incubated with α-m/M, Dox/M and α-m-Dox/M. After 48 h, we collected cells and performed Rh123 staining according to the manufacturer’s instruction.

Cell cycle was measured by PI staining. Gl261 cells were incubated in serum-free DMEM medium (serum starvation) for 24 h, after which cells were co-incubated with α-m/M, Dox/M and α-m-Dox/M. After 48 h, we collected cells and performed PI staining according to standard protocol.

**Western blot**

After different treatments for 48 h, Gl261 cells were washed and the proteins were collected according to the standard protocols. The concentration of total protein was measured by the BCA protein assay kit (Thermo Fisher Scientific, Waltham, MA, USA). Antibodies to Akt (1:1000), phosphor-Akt (1:2000), Bcl-2 (1:1 000), Bax (1:1000), caspase 3 (1:1000), cleaved caspase 3 (1:1000), caspase 8 (1:1000), cleaved caspase 8 (1:1000), caspase 9 (1:1000), cleaved caspase 9 (1:1000), CDK2 (1:1000), CDK4 (1:1000), cyclin D1 (1:1000), cyclin E1 (1:1000), FoxO1 (1:1000), phosphor-FoxO1 (1:1000) (p-FoxO1), p21 (1:1000), p53 (1:1000), GAPDH (1:1000) were used.

**Scratch assay**

A total of 5×10^5^ HUVECs were incubated in medium, transferred into each well of 6-well plates and allowed to form a confluent cell monolayer. When the cell confluence had reached approximately 80%, a sterile yellow tip was used to create a straight line followed by washing cells with phosphate-buffered saline (PBS) to remove the debris, with the cells recovering by adding the serum‐free medium for further culturing. Cells were imaged at the time of 0 h and 48 h by an inverted light microscope (Olympus, Tokyo, Japan). The migration areas were measured by ImageJ software.

**Transwell assay**

HUVECs were treated with DMEM medium for 12 h. Then, the cells were harvested and washed using PBS two times followed by resuspension in a DMEM medium with the cell density having been adjusted to 3 × 10^5^ cells/ml. Then, 200 μl cell suspension was added into the apical chambers, while the basolateral chambers were added with α-m, Dox/M and α-m-Dox/M dissolved in 600 μl DMEM containing 10% FBS. After a conventional culture for 24 h, the Transwell chambers (8 μm, Millipore) were subsequently taken out with the cells on the inner side of apical chambers being wiped out using a cotton swab. The samples were fixed in methanol for 30 min, and stained using 0.1% crystal violet for 20 min. Five visual fields were selected on a random basis under a microscope with pictures taken. The number of cells that migrated through the membrane was counted.

**Tube formation**

Matrigel was thawed at 4°C a day before experiment with the pipette being pre‐cooled at 4°C. Transfer 120 μl of pre-cooled matrigel to a 48‐well plate to solidify at 37°C for 30 min. Collect the primary HUVECs, count and dilute to 4×10^5^ cells/ml in cell culture media. Transfer 110 µl of each diluted cells to 110 µl cell culture media containing α-m/M, Dox/M and α-m-Dox/M, respectively. Mix and transfer 200 µl mixture to the above 48-well plate, which was followed by a 3 h culture in an incubator at 37°C. Five visual fields were selected on a random basis under a microscope with pictures taken. The data were measured by ImageJ software.

**Experiments protocols**

FLK-1 promoter EGFP transgenic (Tg(FLK-1:EGFP)) zebrafish line was used to establish a tumor xenograft zebrafish model. Zebrafish embryos at forty-eight postfertilization (hpf) were stripped off the egg sheath and anesthetized. Then, 300 Gl261 cells labeled with red fluorescent dye (CM-DiI) were injected into zebrafish (the perivitelline space) using a Cell Tram Vario injector equipped with a glass micropipette (L ¼ 50 mm, diameter of the needle opening about 25 mm) under a Zeiss Stemi 2000-C dissecting microscope (Carl Zeiss Microimaging Inc., Thornwood, NY). MPEG-PCL (1 μg/mL), α-m/M (1 μg/mL), Dox/M (1 μg/mL) and α-m-Dox/M (1 μg/mL) were added to the incubating Holtfreter's solution at 24 h after injection of Gl261 cells. On the fifth day after inoculation, images were taken under a confocal microscope.

The murine subcutaneous tumor models were established by subcutaneous injection of 1×10^6^ Gl261 cells per C57BL/6 mice and 1×10^7^ C6 cells per BALB/c nude mice. To establish orthotopic implantation glioma model, 1×10^3^ Gl261 cells suspended in 5 μl PBS was intracranially injected into mice right striatum using a 10 μl Hamilton micro-syringe (Hamilton, Darmstadt, Germany). Before intracranial implantation, C57BL/6 mice were anesthetized by intraperitoneal injection of pentobarbital sodium (80 mg/kg), and then immobilized in a Kopf stereotaxic instrument. Injection into the right cerebral hemisphere (2 mm right to the sagittal suture, 1.5 mm anterior to the coronal suture, 3 mm deep into brain) of Gl261 cells was carried out. After glioma cells inoculation, mice were randomly divided into five groups and named as NS (treatment with normal saline), vehicle (treatment with MPEG-PCL), α-m/M (treatment with α-m/M), Dox/M (treatment with Dox/M) and α-m-Dox/M (treatment with α-m-Dox/M). There were sixteen mice each group, ten of which were used for survival analysis. In vivo, the dosage of Dox/M is 3 mg/kg and α-m/M is 3 mg/kg. For orthotopic implantation model of Gl261, mice were treated on the fifth day after inoculation intravenously once every three days for two weeks. For subcutaneous models of Gl261 and C6, mice were treated every three days once the tumor volume was about 100 mm^3^ and tumor volume of each mice was measured twice a week by a caliper. The weight of mice was measured twice a week. The survival time of each mouse in each group was recorded and Kaplan–Meier survival curve was drawn. The remaining mice were used for in vivo imaging on the 14th day after therapies, and then sacrificed for other experiments.

**Optical in vivo imaging**

In vivo imaging experiment was performed to observe therapeutic effect of drugs. Gl261 cells expressing a transfected luciferase gene had been constructed before, which allows us to monitor tumor engraftment by bioluminescence imaging technology. After treatment of 14 days, mice injected luciferase substrate intraperitoneally were taken images using IVIS Lumina imaging system (Caliper, USA).

**TUNEL assay**

Slides of tumor tissues were performed a classic TUNEL assay to detect and quantitate apoptotic cells according to manufacturer’s instructions. Five visual fields per TUNEL-stained slide were randomly selected for observation and photography under a fluorescence microscope (Leica, Wetzlar, Germany). Image J software was applied to calculate and analyze the density of apoptotic cells and average apoptotic index in each group.

**Histology**

Tumor tissues were taken from the sacrificed mice and fixed in 4% paraformaldehyde for at least 24 h. Paraffin sections of tumor tissues were prepared according to the standard procedure. H&E staining was performed in accordance with previous methods. In addition, immunohistochemical staining of CD31(1:50) and Ki67 (1:50) were carried out to examine angiogenesis and proliferation of tumor. Detailed staining methods followed standard protocols. Five visual fields per slide were randomly selected for photograph under a microscope. The density of positive cells was analyzed by ImageJ software.

**Drug toxicity assessment**

Blood from mice was used for complete blood count (CBC) and serological biochemistry analysis. The blood was kept in 4 ℃ refrigerator overnight and then serum collected by centrifugation at 4 ℃ for 15 min was used to perform serum biochemical analysis by an automatic analyzer (Roche, Basel, Switzerland). CBC was executed using an automatic hematology analyzer (Nihon Kohden, Tokyo, Japan). Organs of heart, lung, liver, kidney and spleen from mice were used for H&E staining.
