# Supplementary material for: Synergetic therapy of glioma mediated by a dual delivery system loading α-mangostin and doxorubicin through cell cycle arrest and apoptotic pathways
Source: Cell Death Dis. 2020 Oct 28;11(10):928. doi: 10.1038/s41419-020-03133-1 (PMC7595144; doi:10.1038/s41419-020-03133-1)
Supplement: Supplementary file 2 — Supplementary figure legends [file 41419_2020_3133_MOESM2_ESM.doc]

**Supplementary Data**

**Supplementary Figure Legends**

**Fig. S1 Computational simulation analysis.** Interaction modes of MPEG-PCL copolymer, α-m and Dox revealed by Langevin dynamics simulation. **a** The initial conformation of MPEG-PCL copolymer complexed with α-m and Dox. The left, the upper right and the lower right represent MPEG-PCL copolymer, α-m and Dox. Two terminal heavy atoms in the MPEG-PCL copolymer are highlighted using a “ball” style. Conformations (**b**), (**c**), (**d**), (**e**) and (**f**) correspond to snapshots of the nanocomposite collected at 100 ps, 200 ps, 300 ps, 400 and 500 ps, respectively.

**Fig. S2 Cell viability study of the nanocomposites by MTT assay.** U87 cells were treated with α-m/M, Dox/M and α-m-Dox/M at different concentrations for 24 h, 48 h and 72 h. The concentrations of the nanocomposites were 0, 0.039, 0.078, 0.156, 0.312, 0.625, 1.25, 2.5, 5 and 10 μg/ml. Error bars denote mean ± s.e.m. Data are representative of at least three independent experiments performed in quintuplicate cultures. *p<0.05, **p<0.01, ***p<0.001; ANOVA and Student’s t-test.

**Fig. S3** **Quantitative analysis of Western blots.** The data were calculated by the ImageJ software. Error bars denote mean ± s.e.m. Data are representative of at least three independent experiments performed in triplicate cultures. *p<0.05, **p<0.01, ***p<0.001; ANOVA and Student’s t-test.

**Fig. S4 Apoptosis assay of C6 cells.** C6 cells were incubated with different nanocomposites at different concentrations for 48 h. Cells were collected and stained with Annexin-V and PI. The concentrations of nanocomposites were 0, 0.039, 0.078, 0.156, 0.312 μg/ml. (**a**) and (**b**) Percentage of apoptotic C6 cells; Error bars denote mean ± s.e.m. Data are representative of at least three independent experiments performed in quintuplicate cultures. *p<0.05, **p<0.01, ***p<0.001; ANOVA and Student’s t-test.

**Fig. S5 Apoptosis assay of U87 cells.** U87 cells were incubated with different nanocomposites at different concentrations for 48 h. Cells were collected and stained with Annexin-V and PI. The concentrations of nanocomposites were 0, 0.039, 0.078, 0.156, 0.312 μg/ml. (**a**) and (**b**) Percentage of apoptotic U87 cells; Error bars denote mean ± s.e.m. Data are representative of at least three independent experiments performed in quintuplicate cultures. *p<0.05, **p<0.01, ***p<0.001; ANOVA and Student’s t-test.

**Fig. S6** **Migration assay and tube formation of HUVECs.** **a** Representative images of HUVEC migration evaluated by scratch assay after 0 h and 48 h. The percentage of cell migration was equal to scratch area of 0 h minus scratch area of 48 h, and then divided by scratch area of 0 h. **b** Representative images of HUVEC migration evaluated by transwell assay after 24 h. **c** The primary HUVEC angiogenesis evaluated by tube formation assay after 3 h. The data were calculated by the ImageJ software. Error bars denote mean ± s.e.m. Data are representative of at least three independent experiments performed in triplicate cultures. *p<0.05, **p<0.01, ***p<0.001; ANOVA and Student’s t-test.

**Fig. S7 Antitumor effect in zebrafish tumor models.** Images of the Gl261 transgenic zebrafish treated with NS, vehicle, α-m/M, Dox/M and α-m-Dox/M, respectively. Data are representative of at least three independent experiments. n=5.

**Fig. S8** **Ki67 staining of tumor tissue sections.** The tumor tissues were from orthotopic implantation glioma mice. **a** Images of Ki67 staining sections. **b** The average percentage of Ki67 positive cells. Error bars denote mean ± s.e.m. Data are representative of at least three independent experiments performed in quintuplicate cultures. *p<0.05, **p<0.01, ***p<0.001; ANOVA and Student’s t-test.

**Fig. S9 Toxicity assay of vital organs with H&E staining.** Main organs of heart, liver, spleen, lung and kidney were collected from mice for histology examination. Data are representative of at least three independent experiments; n=5.

**Fig. S10 Serological biochemical analysis.** Error bars denote mean **±** s.e.m. Data are representative of at least three independent experiments. n=5. Abbreviations: ALB, albumin; ALP, alkaline phosphatase; ALT, alanine aminotransferase; AST, aspartate aminotransferase; AMY, amylase; CK, creatine kinase; CREA, creatinine; GLU, glucose; HDL, high-density lipoprotein-cholesterol; LDL, low-density lipoprotein-cholesterol; LDH, lactate dehydrogenase; TBIL, total bilirubin; TC, total cholesterol; TG, triglycerides; TP, total protein; UA, uric acid; UREA, urea. The horizontal lines indicate the reference range for each analyte. The reference ranges for ALB, ALP, ALT, AST and AMY were 30–42 U/L, 51–159 U/L, 18–98 U/L, 208–380 U/L and 760–3700U/L, respectively. The reference ranges for TC, CK, UREA, CREA and TBIL were 1.82–5.04 mg/dL, 302–960U/L, 4.08–14.28 mmol/L, < 48.2 μmol/L and 5.12–15.8 μmol/L, respectively. The reference ranges for GLU, HDL, LDL, LDH and TG were 3.86–10.22 mmol/L, >0.76 mmol/L, <1.32 mmol/L, 270––1320 U/L and 1.16–4.58 mmol/L, respectively. The reference ranges for TP and UA were 62–86 g/L and <9.5 mg/dL, respectively.

**Fig. S11** **Blood routine examination.** Error bars denote mean **±** s.e.m. Data are representative of at least three independent experiments. n=5. Abbreviations: HGB, hemoglobin; PLT, platelet; RBC, red blood cell; WBC, white blood cell. The horizontal lines indicate the reference range for each analyte. The reference ranges for WB, RBC, PLT and HGB were 6.89–11.25×10^9^/L, 7.6–10.5×10^12^/L, 620–968×10^9^/L and 158–223 g/L, respectively.
