# Supplementary figures and images for: Synergetic therapy of glioma mediated by a dual delivery system loading α-mangostin and doxorubicin through cell cycle arrest and apoptotic pathways
Source: Cell Death Dis. 2020 Oct 28;11(10):928. doi: 10.1038/s41419-020-03133-1 (PMC7595144; doi:10.1038/s41419-020-03133-1)

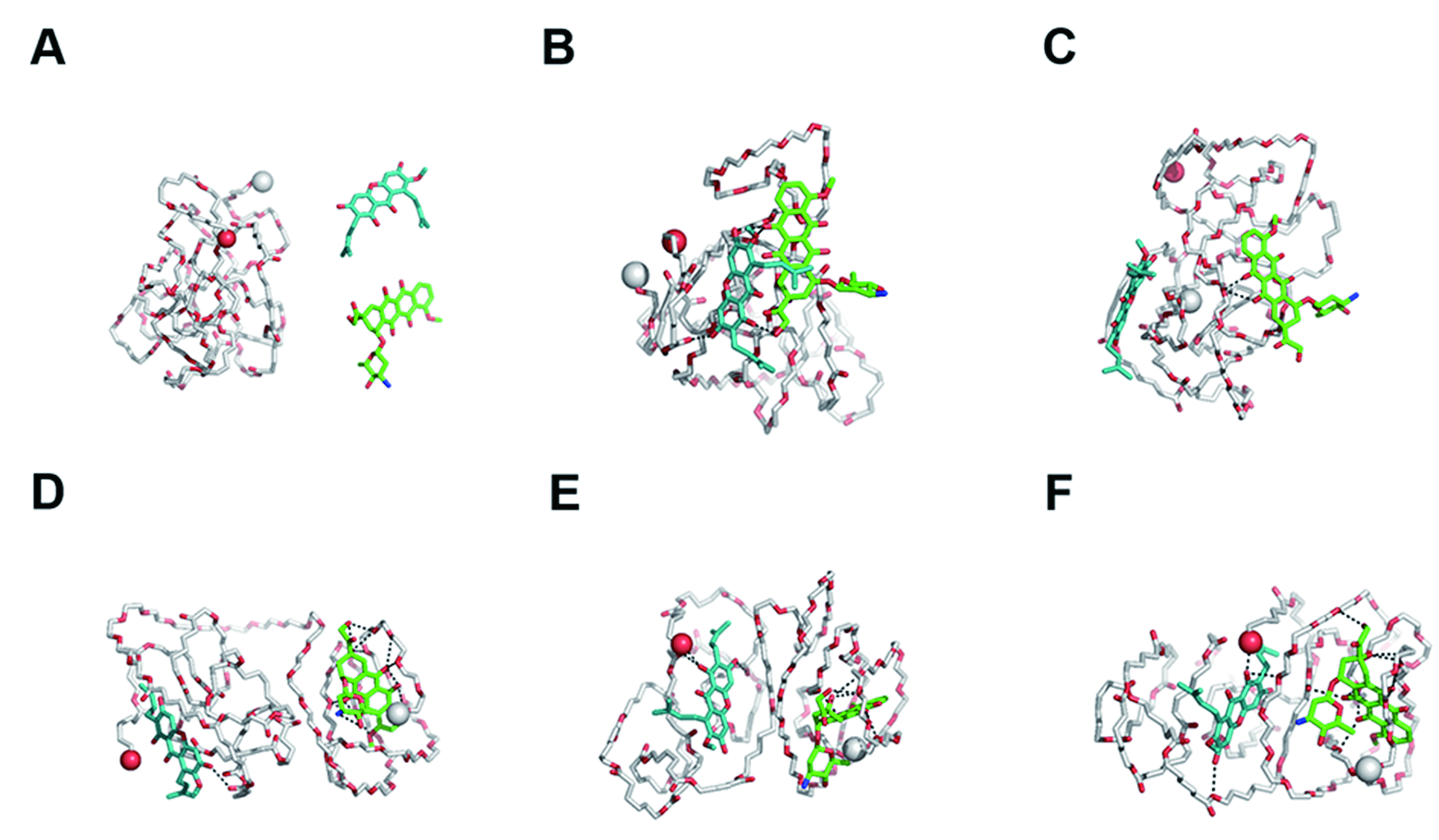

Supplement: Supplementary file 3 — Supplementary figure 1 [file 41419_2020_3133_MOESM3_ESM.tif]

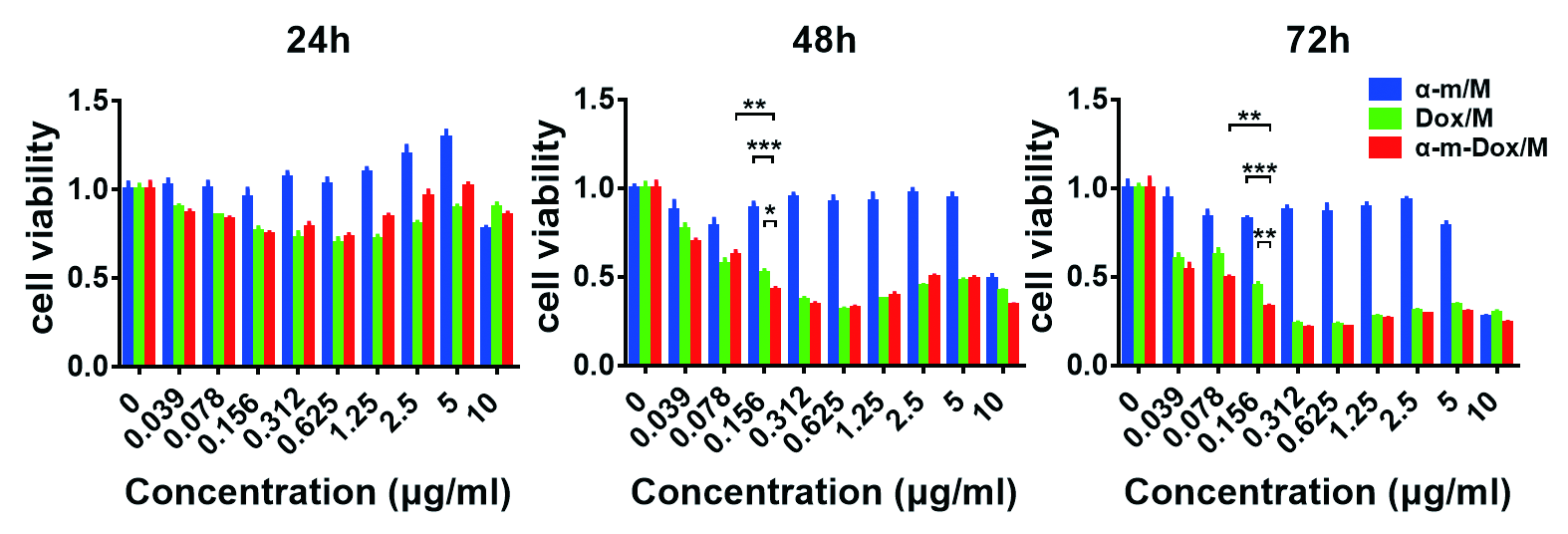

Supplement: Supplementary file 4 — Supplementary figure 2 [file 41419_2020_3133_MOESM4_ESM.tif]

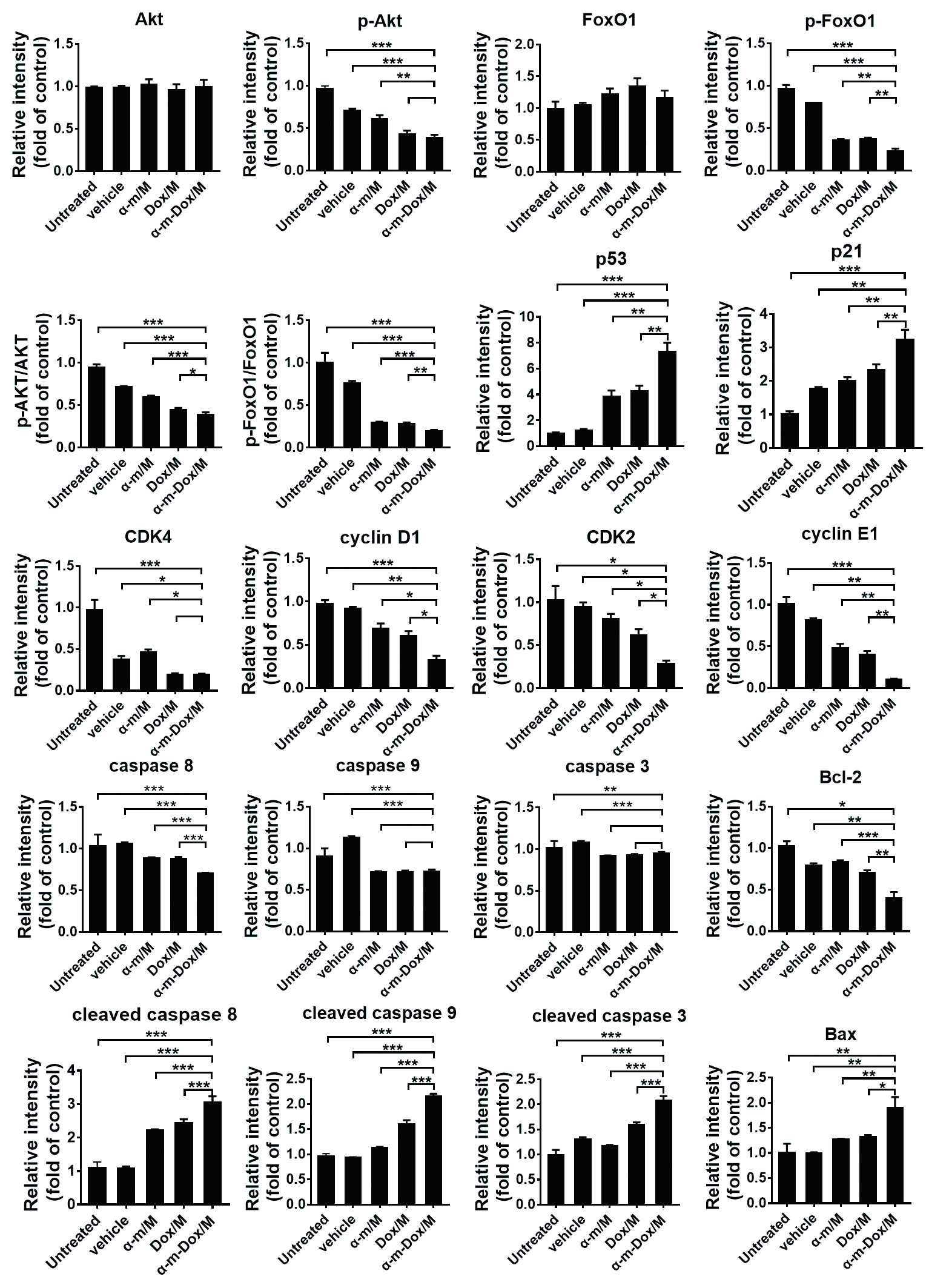

Supplement: Supplementary file 5 — Supplementary figure 3 [file 41419_2020_3133_MOESM5_ESM.tif]

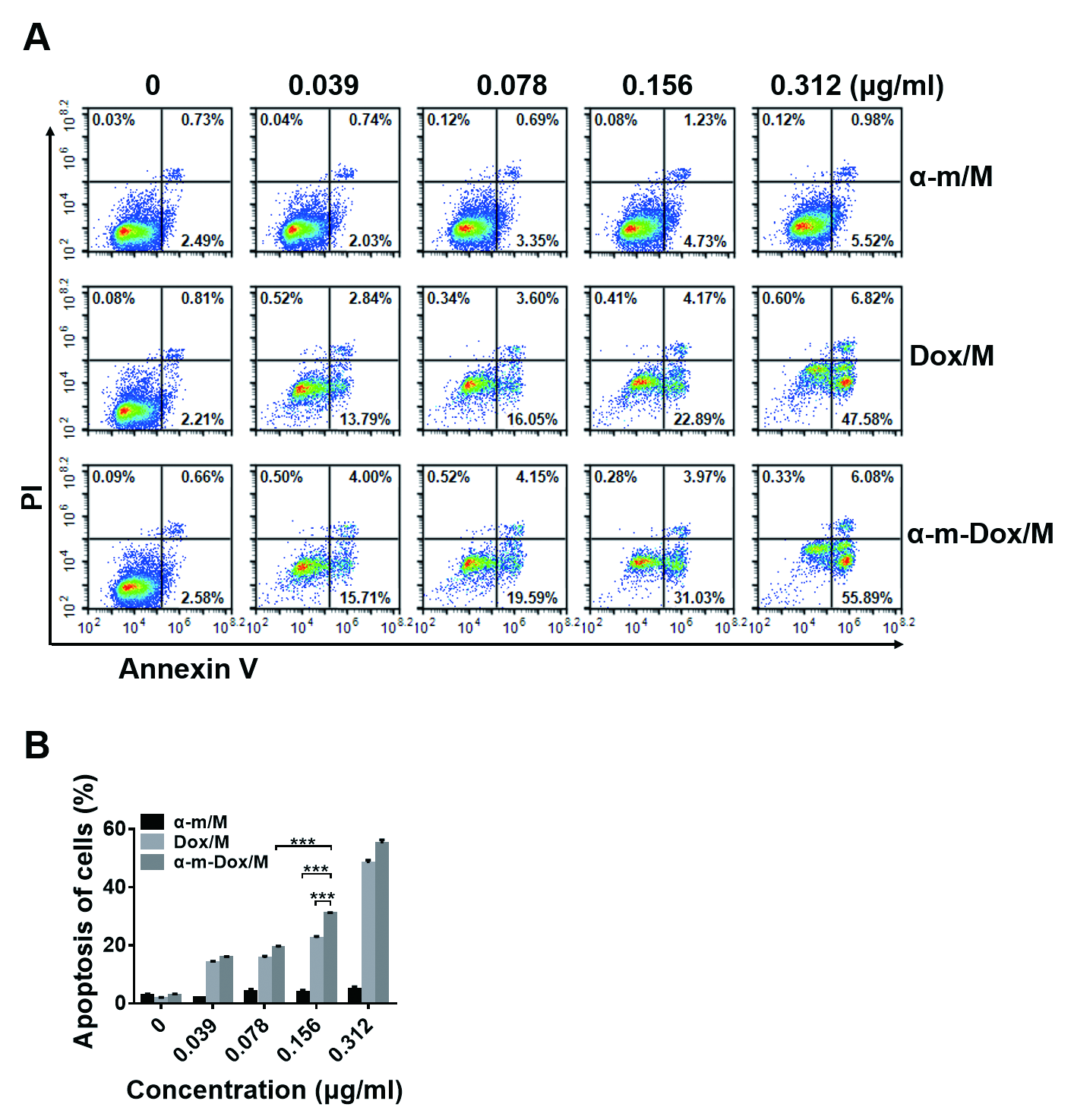

Supplement: Supplementary file 6 — Supplementary figure 4 [file 41419_2020_3133_MOESM6_ESM.tif]

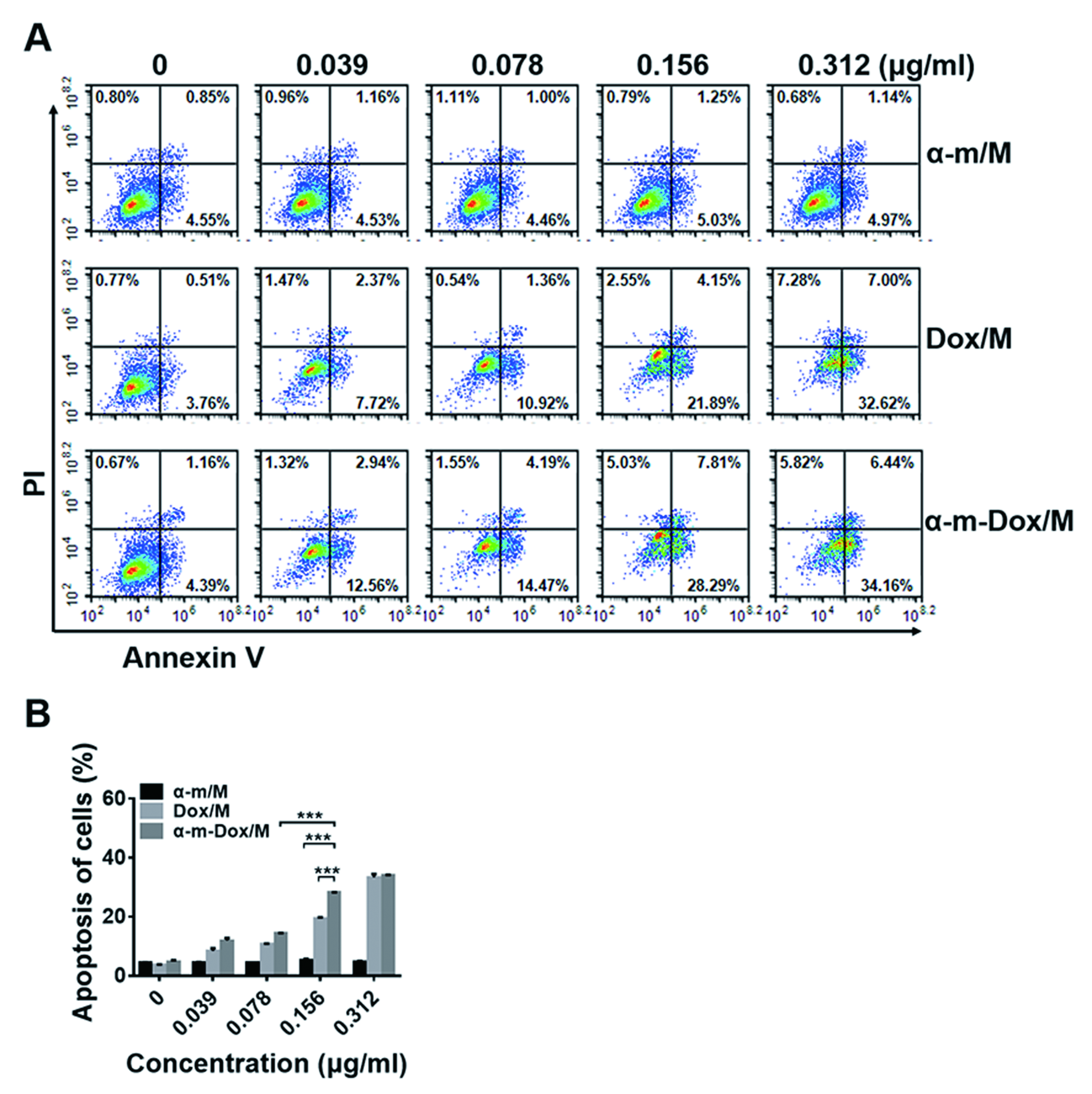

Supplement: Supplementary file 7 — Supplementary figure 5 [file 41419_2020_3133_MOESM7_ESM.tif]

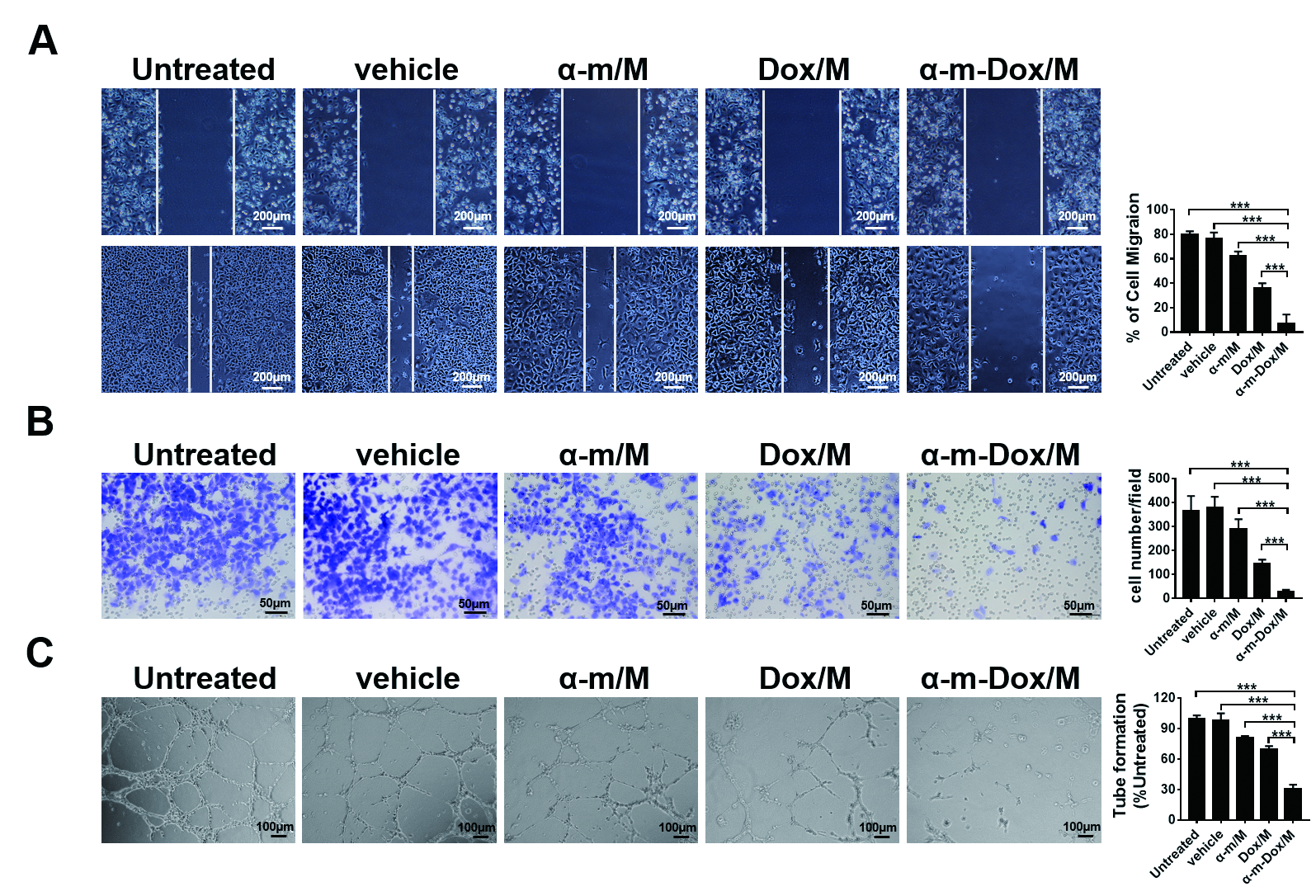

Supplement: Supplementary file 8 — Supplementary figure 6 [file 41419_2020_3133_MOESM8_ESM.tif]

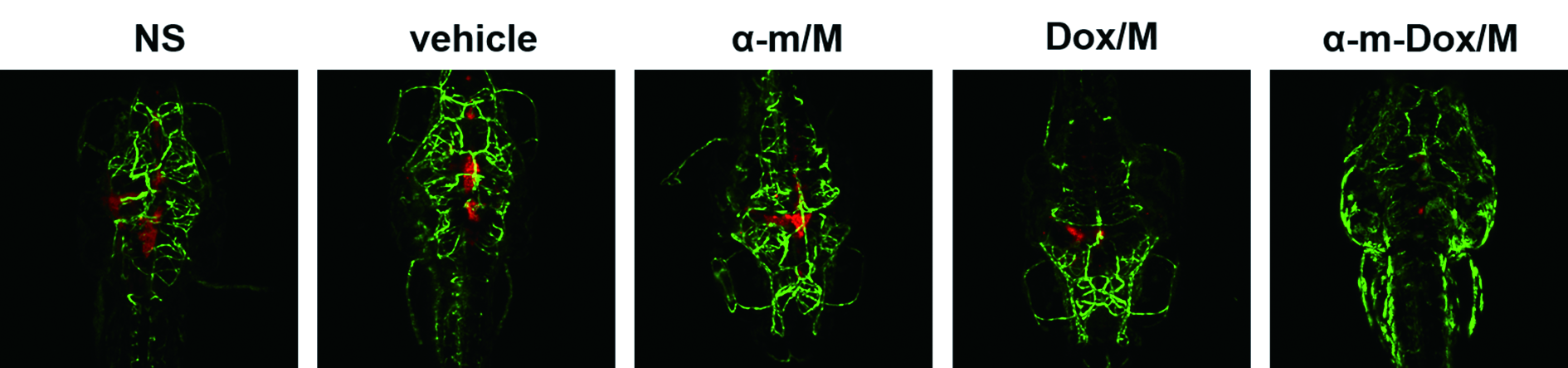

Supplement: Supplementary file 9 — Supplementary figure 7 [file 41419_2020_3133_MOESM9_ESM.tif]

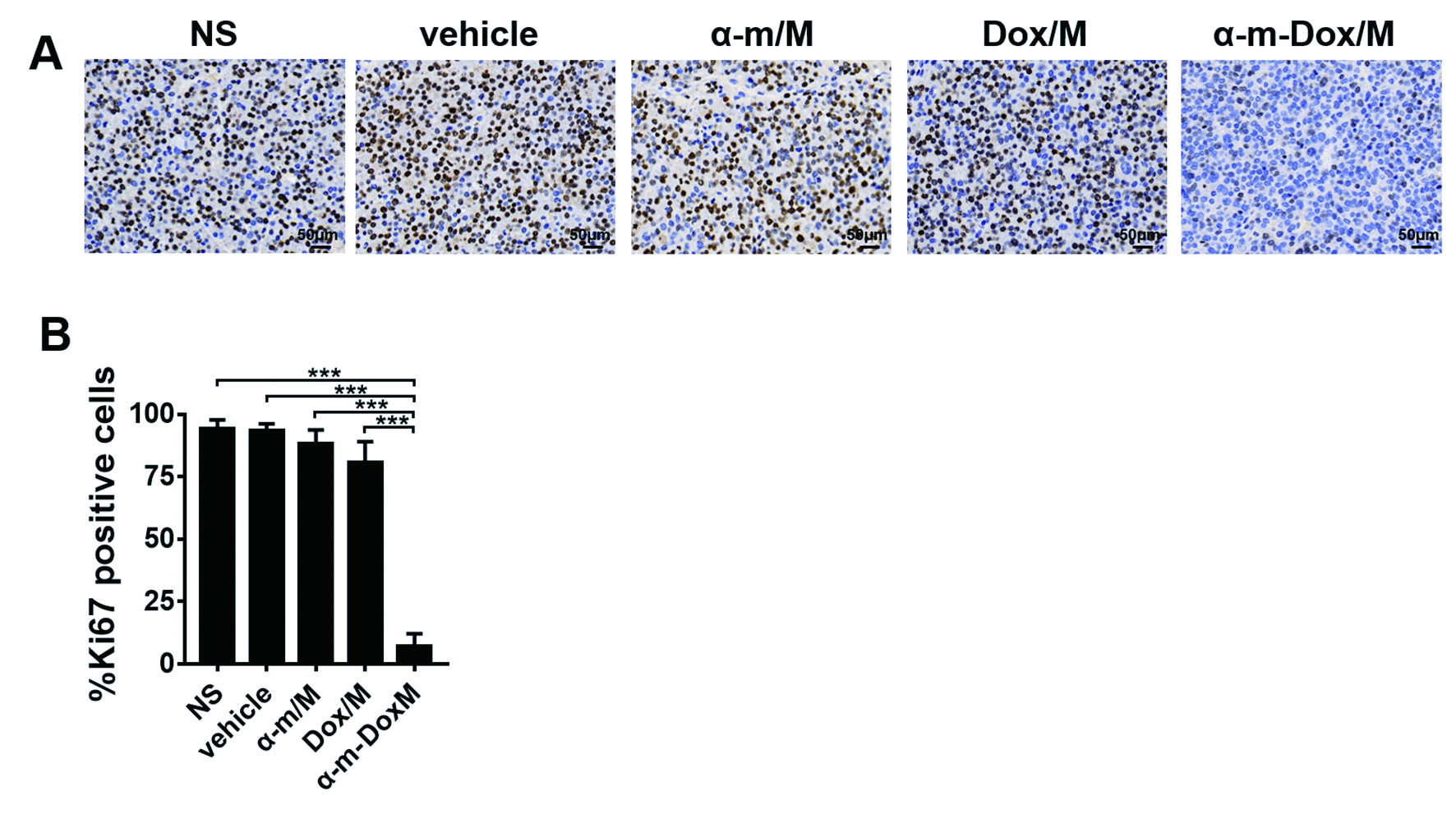

Supplement: Supplementary file 10 — Supplementary figure 8 [file 41419_2020_3133_MOESM10_ESM.tif]

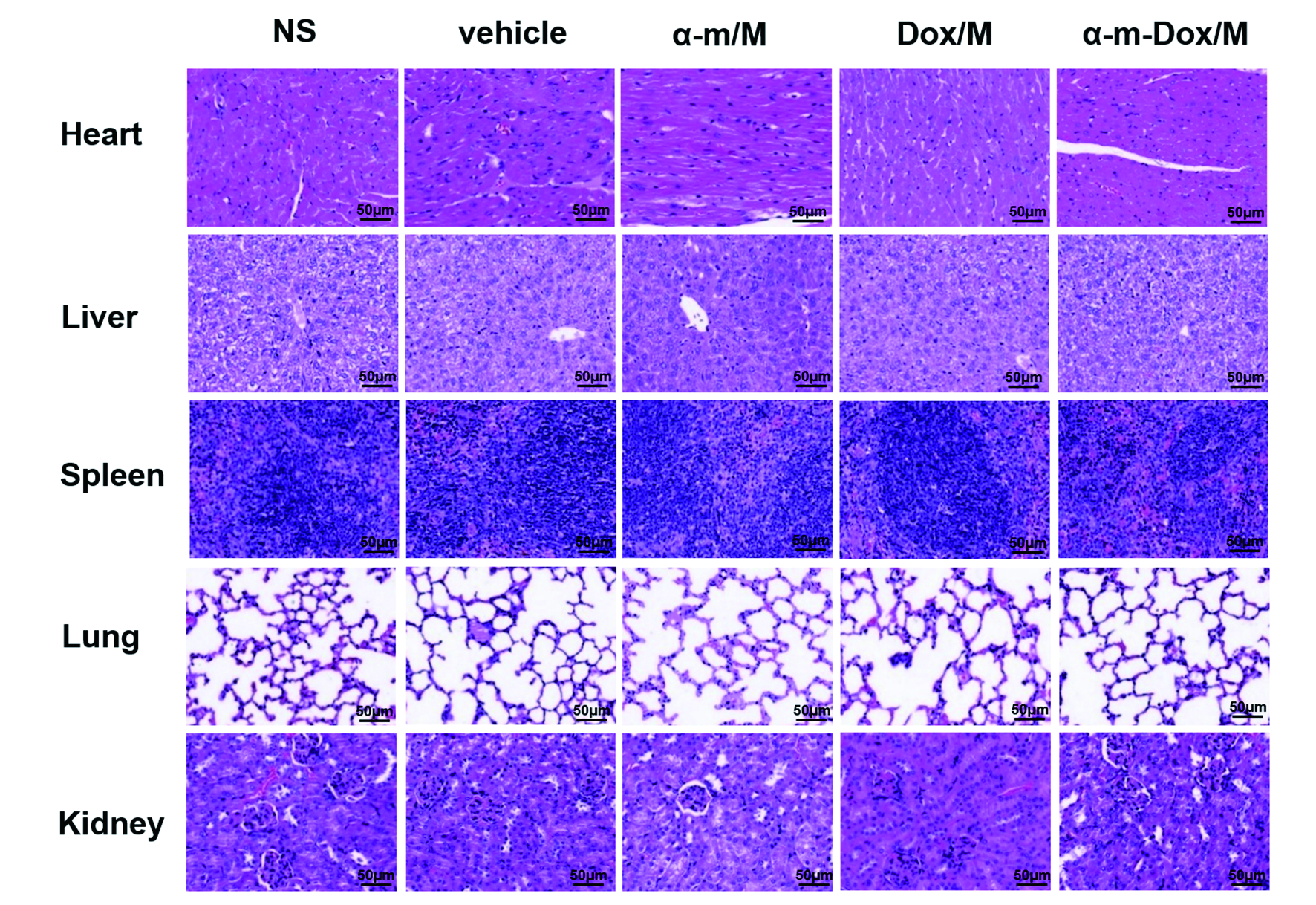

Supplement: Supplementary file 11 — Supplementary figure 9 [file 41419_2020_3133_MOESM11_ESM.tif]

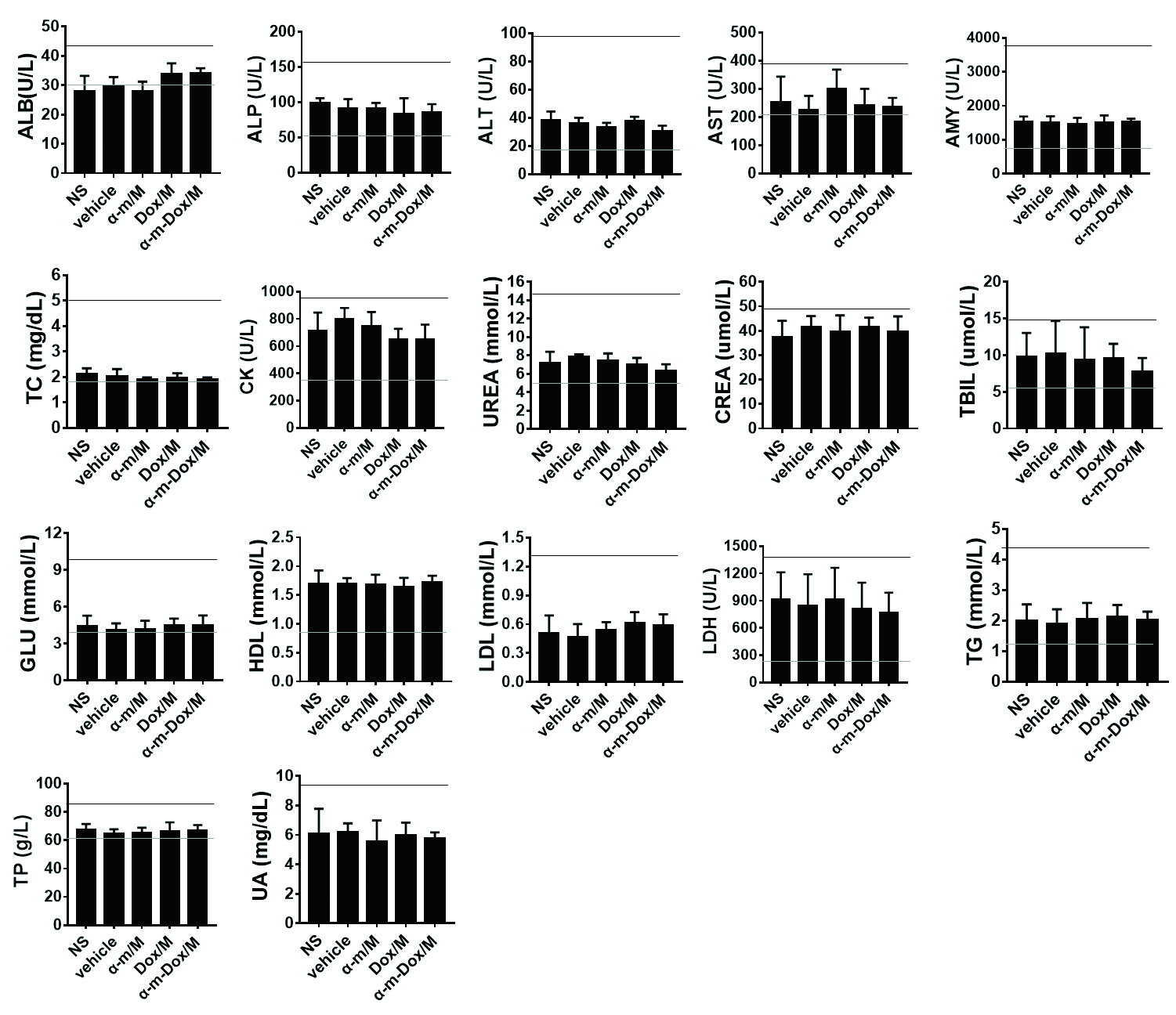

Supplement: Supplementary file 12 — Supplementary figure 10 [file 41419_2020_3133_MOESM12_ESM.tif]

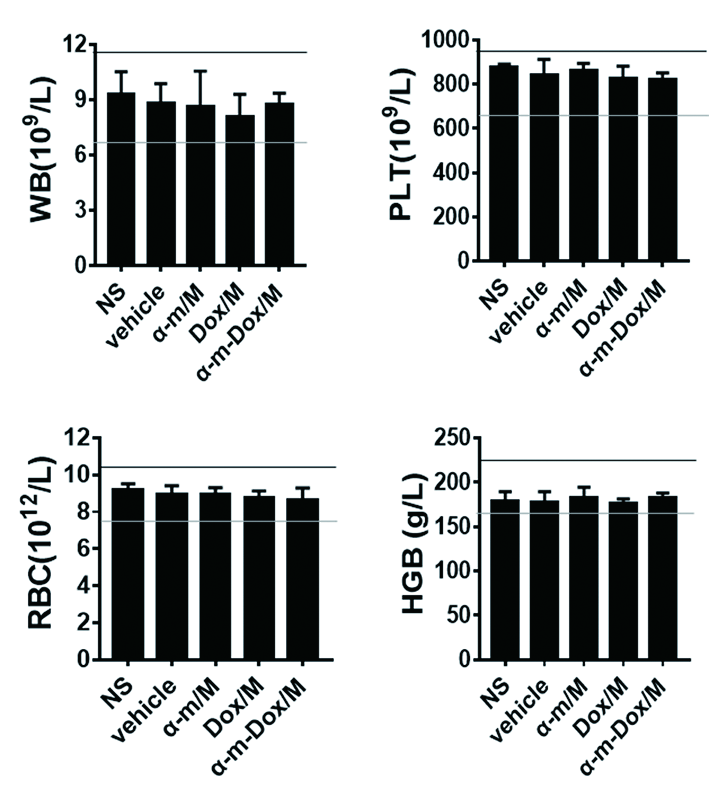

Supplement: Supplementary file 13 — Supplementary figure 11 [file 41419_2020_3133_MOESM13_ESM.tif]
